# Deficiency of Toll-like receptor 2 is a driver of sex-related compositional and structural rearrangements of membrane lipids

**DOI:** 10.1038/s42004-025-01766-x

**Published:** 2025-12-06

**Authors:** Raluca Sabina Ica, Kristina Mlinac-Jerkovic, Mario Stojanović, Maria Roxana Biricioiu, Borna Puljko, Nikolina Maček-Hrvat, Marina Dobrivojević Radmilović, Željka Korade, Karoly Mirnics, David E. Clemmer, Alina D. Zamfir, Svjetlana Kalanj-Bognar

**Affiliations:** 1 National Institute for Research and Development in Electrochemistry and Condensed Matter, Timisoara, Romania; 2 Department of Chemistry and Biochemistry, School of Medicine, University of Zagreb, Zagreb, Croatia; 3 Croatian Institute for Brain Research, School of Medicine, University of Zagreb, Zagreb, Croatia; 4 Division of Molecular Biology, Laboratory for Cell Biology and Signalling, Ruđer Bošković Institute, Zagreb, Croatia; 5 West University of Timisoara, Timisoara, Romania; 6 Institute for Medical Research and Occupational Health, Zagreb, Croatia; 7 Department for Histology and Embryology, School of Medicine, University of Zagreb, Zagreb, Croatia; 8 Department of Pediatrics, Biochemistry and Molecular Biology, College of Medicine, University of Nebraska Medical Center, Child Health Research Institute, Omaha, NE USA; 9 Munroe-Meyer Institute for Genetics and Rehabilitation, University of Nebraska Medical Center, Child Health Research Institute, Omaha, NE USA; 10 Department of Chemistry, Indiana University, Bloomington, IN USA; 11 Department of Technical and Natural Sciences, Aurel Vlaicu University of Arad, Arad, Romania

**Keywords:** Lipidomics, Mass spectrometry, Neurochemistry

## Abstract

The behavior and function of membrane microdomains is shaped by the intricate liaison between the most complex glycosphingolipids-gangliosides, cholesterol and specific classes of transmembrane proteins. Toll-like receptor 2 (TLR2), a pattern recognition receptor localized in lipid rafts, is implicated in different membrane-associated events, some of which overlap between gangliosides and TLR2, such as pathogen recognition and neuroinflammation. Aiming to determine whether TLR2 deficiency influences on the composition and arrangement of membrane lipids, we examined cortical tissue of TLR2-deficient and control mice by in-depth glycolipidomic profiling along with transcriptomic analysis of genes involved in ganglioside and cholesterol metabolism. A multi-level experimental approach, including powerful high-resolution mass spectrometry techniques, provided a detailed lipidomic data and elaborate structural characterization of brain gangliosidome and sterolome in TLR2 deficiency. The results demonstrate the presence of distinct brain glycolipidomic and sterol pattern as well as lipid redistribution within the membrane fractions in TLR2 deficiency. In addition, the findings speak in favor of a sex-specific structural and functional partnership of TLR2, gangliosides and cholesterol in the brain tissue that may act as a connection point integrating extracellular stimuli and modulating neuroimmune response in a sex-dependent manner.

## Abstract (web summary)

Membrane microdomains, influenced by the intricate liaison between glycosphingolipids-gangliosides, cholesterol, and specific classes of transmembrane proteins, play crucial roles in pathogen recognition and neuroinflammation. Here, the authors use high-resolution mass spectrometry to reveal that Toll-like receptor 2 (TLR2) deficiency alters brain glycolipidomic and sterol patterns, suggesting a sex-specific interaction between TLR2, gangliosides, and cholesterol that modulates neuroimmune responses.

## Introduction

The fundamental role of biological membranes in vital cellular processes in health and disease has been well documented^1–3^. Properties of the biological lipid bilayers embedding specific proteins and lipids, their diverse specialized structural and compositional profiles and dynamics of functionally organized membrane domains (lipid rafts, LRs) have been extensively studied^4–6^. Still, the research dealing with cross-talk of select membrane lipids and proteins in the mammalian nervous system remains particularly compelling. The reason for that lies in the fascinating structural intricacy and immense surface of the membranes wrapping neurons and glial cells and the fact that membranes are the site of some of the most elaborate and regulated molecular events related to highly complex brain functions^7^. In this work, we focused on brain gangliosides and sterols as important membrane lipid constituents and investigated their potential relationship with transmembrane protein Toll-like receptor 2 (TLR2).

Gangliosides, complex membrane glycosphingolipids markedly abundant in the central nervous system, are essential in processes related to neurodevelopment and neurodegeneration^8–12^. In addition, they participate in tumorigenesis, susceptibility to infection as well as modulation of the immune response by being targets for certain bacterial toxins and viruses^13–18^. Many of the processes mediated by gangliosides are related to their localization in LRs, membrane domains enriched with glycosphingolipids and cholesterol, considered as cellular signaling hubs. Some of the available data on relationship of gangliosides and cholesterol include: structural and functional partnership of cholesterol and glycosphingolipids in the LRs assembly and membrane arrangement; association of metabolic processes where alteration in one of the metabolic pathways influences on another (for ex. disturbed ganglioside synthesis impacts on transcription of genes needed for cholesterol homeostasis); their joint actions during neurodevelopment and neurodegeneration^19–23^. Apparently, gangliosides stand out as pivotal players in processes related to LRs, as supported by studies that established the effects of altered expression of specific gangliosides on proper positioning and functions of transmembrane proteins, particularly the ones involved in synaptic plasticity and maintenance of ion homeostasis^24,25^. Further, the most recent research employing advanced methodological strategies confirmed high structural diversity and distinct spatial distribution of gangliosides in the mammalian brain, and identified specific interactions with transmembrane proteins mostly engaged in cell-adhesion and signal transduction^24,26^.

Of particular interest for this study is the previously reported functional association of gangliosides GD1a and GT1b with TLR2, another LR resident, justifying the role of gangliosides as modulators of immune response^27,28^. Many investigations have so far focused on immunity-related TLR2 functions, however the tasks of TLR2 in the brain seem to be much more complex as shown by studies indicating its specific expression and actions during neurodevelopment^29–33^. Generating the genetically modified murine model with TLR2 deficiency greatly helped in deciphering TLR2 physiological functions, but there are still many gaps in describing the biochemical phenotype of TLR2-deficient mice (TLR2D) or understanding the consequences of TLR2-deficiency for other cellular/membrane/molecular systems.

Having in mind previously depicted shared roles of gangliosides and TLR2 related to inflammatory signaling in the brain, functional association of TLR2 with specific gangliosides, adjustments of cholesterol metabolism in immune response via TLR2 signaling, and localization of transmembrane TLR2 in lipid rafts, we hypothesized that TLR2 deficiency may have an impact on the composition and arrangement of membrane lipids^27,28,34,35^. Aiming to assess a detailed lipidomic profile of brain tissue samples derived from mice with TLR2 deficiency and the sex/age-matched controls, we utilized a multi-scale experimental approach that encompassed transcriptomic and analytical chemistry methods. The latter ones included techniques with evidenced huge potential in providing valuable compositional and structural data for the identification of relevant (patho)physiological biomarkers: liquid chromatography (LC) mass spectrometry (MS) and highly-sensitive advanced MS approaches, such as ion mobility separation (IMS) combined with MS^36–39^. Our results confirmed that TLR2 deficiency is accompanied by a distinct brain glycolipidomic and sterol pattern. Another intriguing finding of the study corroborates the sex-dependent compositional changes of gangliosides and sterols in brain tissue driven by TLR2 deficiency, as substantial specific differences in explored transcriptomic and lipidomic profiles were determined by comparing male and female controls with TLR2D male and female mice.

## Results

### Brain glycolipidomic pattern in TLR2 deficiency is characterized by high molecular complexity and diversity of glycan moiety of gangliosides

Separation of brain gangliosides by high performance thin layer chromatography (HPTLC) revealed the presence of all major ganglioside species (GM1, GD1a, GD1b, GT1b and GQ1b) in cortical tissue samples of both TLR2D and control mice, while quantitative analysis determined a lower total sialic acid concentration in male and female TLR2D mice in comparison with controls (Supplementary Fig. 2). Although the differences in sialic acid concentration in brain tissue samples between compared groups of experimental animals did not reach statistical significance, the results indicated that a lower amount of gangliosides is sex-related, i.e., a feature of both control and TLR2D female mice when compared with control and TLR2D male mice (Supplementary Fig. 2C).

**Fig. 1:**
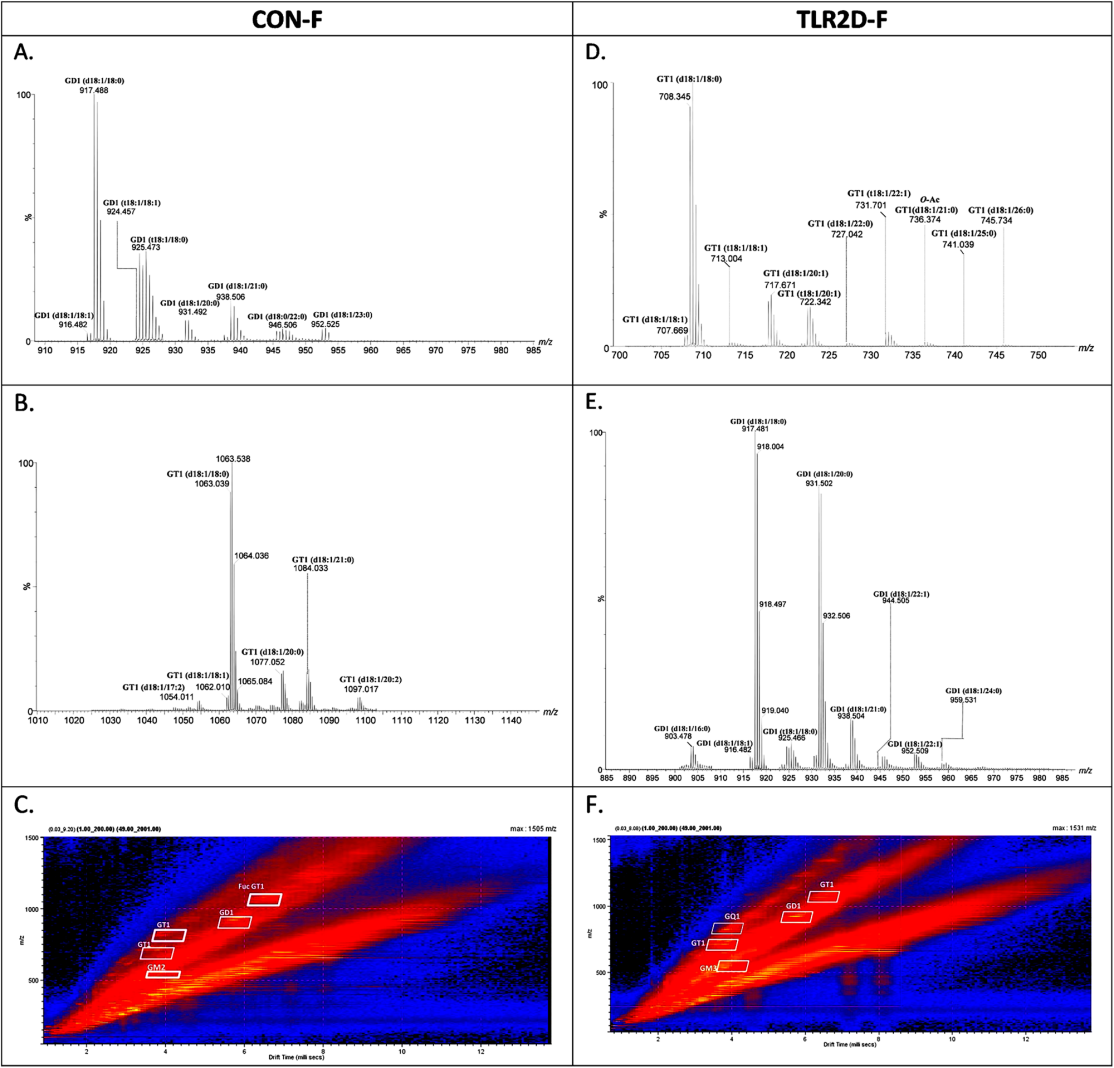
Extracted mass spectra of disialo- and trisialo-gangliosides species present in brain tissue of TLR2 deficient and control female mice. **A** doubly charged GD1 and **B** doubly charged GT1 from the corresponding areas indicated in **C** Driftscope display (drift time versus *m/z*) showing the total distribution of electrosprayed GG ions in brain samples derived from control female mice (CON F); **D** triply charged GT1 and *O*-Ac GT1, **E** doubly charged GD1 from the corresponding areas indicated in **F** Driftscope display (drift time versus *m/z*) showing the total distribution of electrosprayed GG ions from brain samples of TLR2 deficient female mice (TLR2D F). The GG separation in the drift cell was according to the charge state, carbohydrate chain length and the degree of sialylation.

**Fig. 2:**
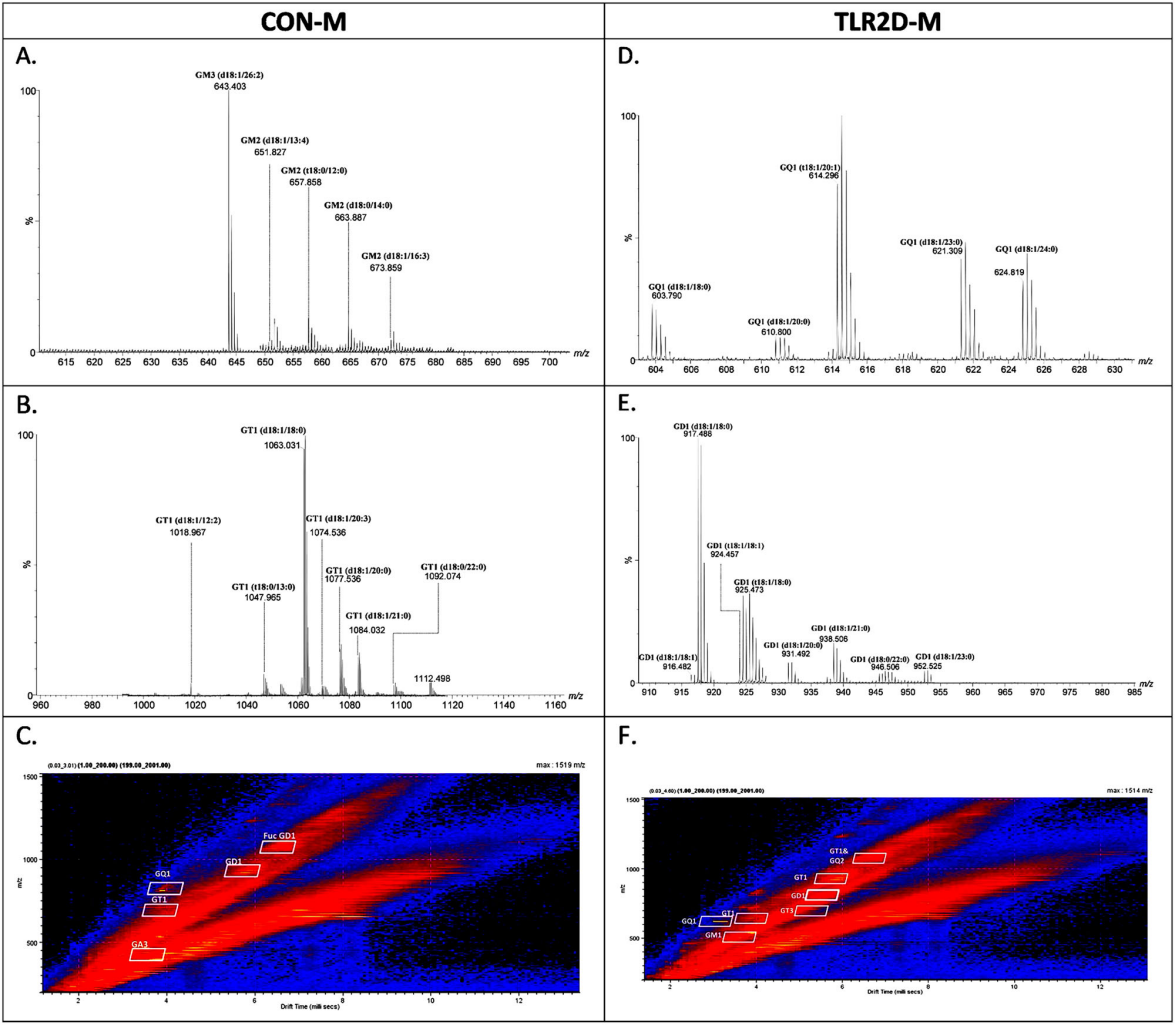
Extracted mass spectra of monosialo-, disialo- and tetrasialo-ganglioside species present in brain tissue of TLR2 deficient and control male mice. **A** Doubly charged GM2 and GM3, **B** doubly charged GD1 from the corresponding areas indicated in (**C**) Driftscope display (drift time versus *m/z*) showing the total distribution of electrosprayed GG ions in brain samples derived from control male mice (CON M); **D** quadruply charged GQ1, **E** doubly charged GD1 from the corresponding areas indicated in **F** Driftscope display (drift time versus *m/z*) showing the total distribution of electrosprayed GG ions from brain samples of TLR2 deficient male mice (TLR2D M). The GG separation in the drift cell was according to the charge state, carbohydrate chain length and the degree of sialylation.

On the other hand, subsequent structural characterization of ganglioside species performed by a more potent and sensitive nano-electrospray ionization mass spectrometry (nanoESI-IMS) method detected a remarkably higher diversity and molecular complexity of brain ganglioside species in TLR2D mice of both sexes when compared with controls. The driftscope displays, which present the total distribution of gangliosides and the mass spectra generated by integrating the two-dimensional data across all *m/z* values and drift times, are shown in Figs. 1 and 2. The driftscope plots demonstrate that impurities and contaminants inherently present in a biological extract were clearly delineated, while the different ganglioside species were separated into mobility families based on charge state, glycan core length, and the number of Neu5Ac residues, facilitating the detection of numerous species, including low-abundance glycoforms with biological importance. Figures 1 and 2 present examples of (-) nanoESI IMS mass spectra of relevant singly, doubly, triply, and quadruply charged mono- to tetrasialylated gangliosides, extracted from the areas indicated in the driftscope display. More specifically, Figs. 1C, F and 2C, F show the retention of drift times for each specific region, while the extracted ion chromatograms (XICs) enabled generation of mass spectra for: doubly charged GD1 and GT1 in CON F samples (Fig. 1A, B); doubly charged GD1, triply charged GT1 and *O*-Ac-GT1 in TLR2D F samples (Fig. 1D, E); doubly charged GD1 in CON M samples (Fig. 2A, B); quadruply charged GQ1 and doubly charged GD1 in TLR2D M samples (Fig. 2D, E).

**Fig. 3:**
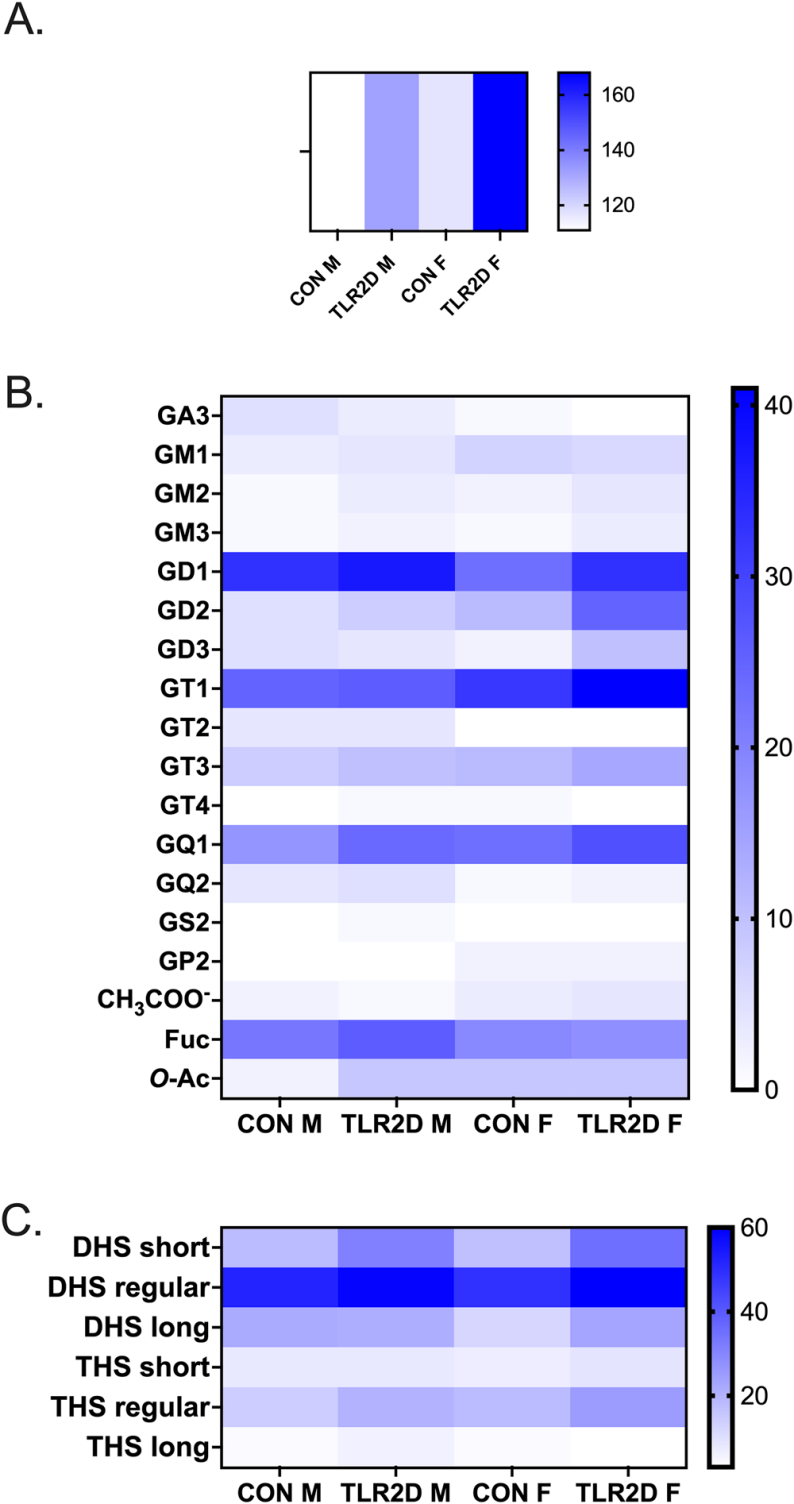
Summarized findings on glycolipidomic analysis of brain tissue derived from mice with TLR2 deficiency in comparison with control mice. **A** Comparison of total and **B** individual number of the identified ganglioside species vs. the composition of their glycan moiety in TLR2-deficient male (TLR2D M) and female mice (TLR2D F) and matched controls (CON M, CON F). **C** Differences in ceramide composition of gangliosides, derived from male and female TRL2 deficient and control mice, regarding the length of fatty acyl chains: short-chain (12–17 carbon atoms), regular-chain (18–22 carbon atoms) and long-chain (24 or more carbon atoms); the dihydroxylated sphingoid bases (DHS) and trihydroxylated sphingoid bases (THS) are designated as DHS-short, DHS-regular, DHS-long and THS-short, THS-regular, THS-long, respectively. Each of the analysis shown in (**A**–**C**) has been performed on tissue samples derived from 3 animals per group.

Interestingly, further structural analysis of brain ganglioside species derived from control and TLR2D mice confirmed characteristic sex-related differences. Considering the abundance of molecules detected in TLR2D F and CON F using negative ion mode ESI-IMS-MS, we have systematically categorized the mainly singly to quadruply deprotonated species corresponding to ganglioside components. This categorization is detailed in Supplementary Table 1, which includes experimental and theoretical *m/z* values corresponding to the proposed structures, along with the calculated mass accuracy. The assignment of ions to ganglioside species was based on precise mass measurements and confirmation by collision-induced dissociation MS (CID MS/MS) of nearly 80% of the structures, with relevant examples illustrated in Supplementary Fig. 3 and 4. As shown, both the TLR2D F and CON F cortical samples exhibit a rich diversity of ganglioside species, characterized by variability in glycan and ceramide structures (Supplementary Table 1). Diversity is evident not only in the body of the glycan, as indicated by the presence of GA3 or GP2 species in both groups of samples, but also in the fatty acid chains of ceramides, which range from 12 to 29 carbon atoms. In terms of glycan diversity, the samples display a range of structures, reflecting different configurations and compositions of sialic acid and other sugar residues. Similarly, the ceramide portion of the gangliosides shows considerable variability in chain length and composition. This variability, spanning from shorter carbon chains to longer ones, suggests diverse lipid environments. To summarize, IMS MS revealed highly complex ionic molecular patterns for CON F and TLR2D F samples, i.e., at least 240 anions with charges ranging from 1 to 4 were detected (Supplementary Table 1). Considerable differences are evident when comparing the abundance of ganglioside species in TLR2D F and CON F samples, as visible in Fig. 3. Specifically, our analysis revealed 168 ganglioside species in the TLR2D F while only 117 in the CON F samples. In the samples from TLR2D F, a larger number of GD1-type species were detected: 33 different GD1 species, compared to 23 GD1 species found in CON F; 25 distinct GD2 species in the TLR2D F and only 11 GD2 species in CON F samples. Similar differences were observed in GD3 (10 species in TLR2D F *vs*. 2 species in CON F) and GT1 species (41 in TLR2D F vs. 32 in CON F).

Regarding structural characterization of gangliosides isolated from cortical samples of male animals, the results encompass a detailed comparative list of the ions detected in TLR2D M and CON M samples, along with their assignments based on precise mass measurements and, in some cases, MS/MS confirmation (Supplementary Table 2). The spectra in Fig. 2, acquired using IMS MS, showcase the effective separation of ganglioside species based on their charge states and sialylation levels. Of note is finding of quadruply charged ions of GQ1-type in TLR2D M samples (Fig. 2D), which were detected neither in CON M nor in TRL2D and CON females. Also, Fig. 2C shows the retention of drift times for each specific region, where the XICs enabled the generation of mass spectra for doubly and triply charged ganglioside classes for CON M samples, while spectra obtained from IMS MS specifically show the doubly charged ions of ganglioside GM2 in CON M and ganglioside GD1 in TLR2D M samples (Fig. 2A, E). In conclusion, in male TLR2D and CON cortical samples, GM gangliosides were detected in both mono- and doubly charged forms, GD components appeared as doubly and triply charged molecules, whereas GT and GQ were found as doubly, triply, and quadruply charged species, among other variations. Comparable to the samples derived from TLR2D F mice, a greater abundance of ganglioside species was determined in TLR2D M when compared with CON M cortical samples (132 species vs. 111, respectively) (Fig. 3A). Regarding the comparison of various classes of gangliosides our results indicate: (a) a notable predominance of polysialylated ganglioside species with extended sugar chains in both TLR2D M and CON M samples; (b) a larger number of GD1, GD2, GT1 and GQ1 species in TLR2D M than in CON M samples (Fig. 3B.). The latter finding is similar to the observation in samples derived from female mice, however the increased presence of specific ganglioside species in TLR2D mice was more pronounced in female than in male animals (Fig. 3B).

### Structural diversity and modifications of brain ganglioside species are expressed in a sex-related manner in TLR2 deficiency

Comparison of the total and individual number of the identified ganglioside species *vs*. the composition of their glycan moiety in TLR2-deficient male and female mice and matched controls shows considerable differences (Fig. 3). This observation speaks in favor of the hypothesized structural rearrangement of membrane lipids due to TLR2 deficiency, which seems to occur in a sex-dependent manner. For instance, the TLR2D M samples exhibit 37 distinct GD1 species, whereas the TLR2D F samples express 33 GD1 species. Conversely, GD2-type species are more abundant in TLR2D F, with 25 species *vs*. only 8 species in TLR2D M. In the TLR2D M and CON M sample groups, no species in the GP-type were detected, contrasting with the identification of this class of gangliosides in the TLR2D F and CON F samples. On the other hand, GS2(d18:1/18:2) forms were specifically identified only in the TLR2D M sample at *m/z* 1041.073 (Supplementary Table 2). Characterization of individual ganglioside components by ESI MS indicates a striking difference even between control male and female mice (Fig. 3B). Specifically, the CON F samples feature by a prevalence of polysialylated species of the GT1 and GQ1 types; in contrast, the CON M samples exhibit the dominance of GD1 and fucosylated structures (Fig. 3B). Additional detailed analysis of ganglioside structural modifications, such as the presence of fucosylation and *O*-acetylation, provided highly useful information for assessing the differences and similarities between male and female TLR2D and CON mice. In females, the similarities between TLR2D F and CON F include the occurrence of common species such as Fuc GT3(t18:0/16:0), Fuc GD1(t18:0/24:0), Fuc GD1(t18:1/24:0), Fuc GD1(d18:1/19:0), and Fuc GT3(t18:0/20:0). Differences are reflected in the diversity and presence of specific species in each sample, for instance, species such as Fuc GM1(d18:1/20:3) and Fuc GM1(d18:1/21:0) are present only in CON F. In contrast, the TLR2D F samples uniquely contain species such as Fuc GD1(t18:1/24:1) and Fuc GT1(d18:1/18:2). The comparison of *O*-acetylated gangliosides between CON F and TLR2D F samples highlights distinct structural patterns and their implications in cellular function. In CON F, species such as *O*-Ac GQ1(t18:0/24:0), *O*-Ac GM1 (d18:0/22:2), and *O*-Ac GQ1 (d18:1/19:0) are identified, and characterized by longer sugar chains indicative of complex glycosylation. Conversely, TLR2D F samples predominantly feature species with shorter sugar chains, including *O*-Ac GM1(d18:1/20:0), *O*-Ac GT3 (d18:0/19:0), and *O*-Ac GT3 (d18:0/24:0). Comparing the composition of the fucose-modified brain glycolipid species in males shows significant differences between the CON M and TLR2D M samples. In the CON M samples, the associated gangliosidome includes various forms of Fuc-GD1, Fuc-GM1, Fuc-GT1, and Fuc-GT3, each with distinct structural modifications such as different fatty acid chain lengths, while in the TLR2D M samples, Fuc-GD1- and Fuc-GT3-modified species predominate, however with an emphasis on species containing shorter glycan chains. Notable differences were revealed also in the composition of the *O*-acetylated ganglioside species expressed in the CON M vs. TLR2D M samples. TLR2D M samples contain a diverse range of *O*-Ac-modified gangliosides, including *O*-Ac GM1 (d18:0/20:2), *O*-Ac GQ1 (d18:1/18:0), *O*-Ac GQ1 (t18:0/17:0), *O*-Ac GT1 (d18:0/22:0), *O*-Ac GT3 (d18:0/22:0). In contrast, CON M are characterized by a much more limited set of *O*-Ac gangliosides, particularly *O*-Ac GQ1 (d18:1/18:0) and *O*-Ac GT3 (t18:0/22:0).

To summarize, the structural characteristics of brain ganglioside species in TLR2-deficient mice include plenty of modifications such as fucosylations and *O*-acetylations, which may specifically discriminate both between males and females, and between TLR2D and control mice (Table 1, Fig. 3).

**Table 1:** Detected modifications of ganglioside species specifically expressed in male and female TLR2-deficient mice in comparison with control mice

	Fucosylated ganglioside species (*Fuc*)	*O*-acetylated ganglioside species (*O-Ac*)
CON F	Fuc-GM1 (d18:1/20:3) Fuc-GM1 (d18:1/21:0)	*O*-Ac GQ1 (t18:0/24:0) *O*-Ac GM1 (d18:0/22:2) *O*-Ac GQ1 (d18:1/19:0)
TLR2D F	Fuc-GD1 (t18:1/24:1) Fuc-GT1 (d18:1/18:2)	*O*-Ac GM1(d18:1/20:0) *O*-Ac GT3 (d18:0/19:0) *O*-Ac GT3 (d18:0/24:0)
CON M	Fuc GD1 Fuc GM1 Fuc GT1 Fuc GT3	*O*-Ac GQ1 (d18:1/18:0) *O*-Ac GT3 (t18:0/22:0)
TLR2D M	Fuc-GD1 Fuc-GT3	*O*-Ac GM1 (d18:0/20:2) *O*-Ac GQ1 (d18:1/18:0) *O*-Ac GQ1 (t18:0/17:0) *O*-Ac GT1 (d18:0/22:0) *O*-Ac GT3 (d18:0/22:0)

CON F and CON M, control female and male mice, TRL2D F and TLR2D M TLR2-deficient female and male mice.

### High heterogeneity of the ceramide moieties composition is an additional structural feature of brain gangliosides in TLR2-deficiency

The analysis of ceramide (Cer) composition of characterized ganglioside species has taken into account the length of the fatty acyl chains, degree of saturation and hydroxylation of the sphingoid bases. For that purpose, we used the following categorization of ceramides: dihydroxylated (DHS) and trihydroxylated (THS) sphingoid bases with short (12–17 carbon atoms), regular (18–22 carbon atoms) and long fatty acyl chains (24 or more carbon atoms). When comparing sample data, the TLR2D F samples contained the highest number of DHS-regular species with 58 components, followed by CON F and TLR2D M with 49 and 59 DHS-regular components, respectively (Fig. 3C). The presence of long-chain fatty acids remains less frequent overall, especially in the THS-long category, with significantly fewer components across all samples. Another noteworthy finding is the high heterogeneity and substantial differences in the composition and structure of the Cer moieties in the ganglioside species expressed in CON F and TLR2D F. Hence, in the CON F sample, species such as Fuc-GD1 (t18:0/24:0) detected as [M-2H^+^]^2-^ and assigned with a mass accuracy of 7.6 ppm, and GD1 (d18:1/24:0) demonstrate great diversity in fatty acid chain length and degree of saturation. In contrast, in the TLR2D F, we observed a different range of gangliosides, such as GT1 (d18:1/26:1), GT1 (d18:1/26:2), and GT1 (d18:1/27:0), which contain longer fatty acid chains. Comparing the ceramide blocks between the CON M and TLR2D M samples, we found that, in general, the length of the fatty acid chains is similar between the two groups of samples, thus we could identify species with short fatty acid chains such as GD1 (d18:1/17:0) and GQ1 (d18:0/17:0), but also species with ceramides whose fatty acid has a long acyl chain such as GD1 (d18:1/25:1) and GT1 (d18:1/24:1).

### IMS CID MS/MS resolved the structure of a GD1b isomer as the most abundant disialylated ganglioside species in brain tissue derived from TLR2-deficient male mice

Several ions listed in Supplementary Tables 1 and 2, particularly those corresponding to potential biomarker species, were analyzed using IMS CID MS/MS to validate their composition and elucidate their detailed structure. The goals of this fragmentation analysis included confirmation of the glycan core configuration, validation of the ceramide composition (including hydroxylation and double bonds), and determination of the Neu5Ac residue localization for identifying the ganglioside isomer.

**Fig. 4:**
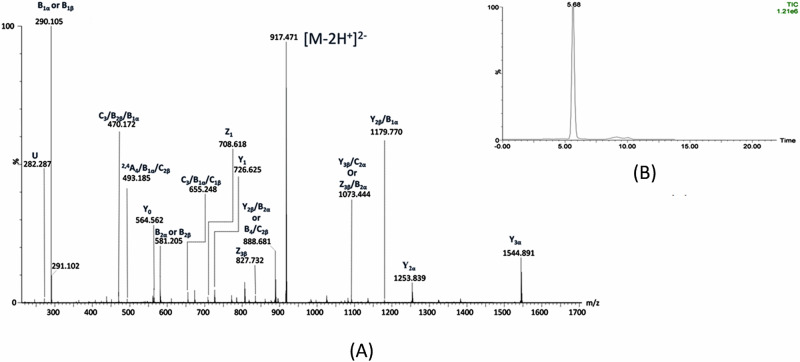
Structural characterization of GD1 isomer, the most abundant disialo-ganglioside species in brain tissue of TLR2-deficient male mice. (-) NanoESI IMS CID MS/MS of the [M-2H^+^]^2-^ at m*/z* 917.488 corresponding to GD1(d18:1/18:0) species detected in the IMS MS of TLR2D-M sample. CID at variable collision energy within (30–60) eV; acquisition 150 scans (**A**); drift time distribution for the ion at *m/z* 917.488 fragmented by CID (**B**).

**Fig. 5:**
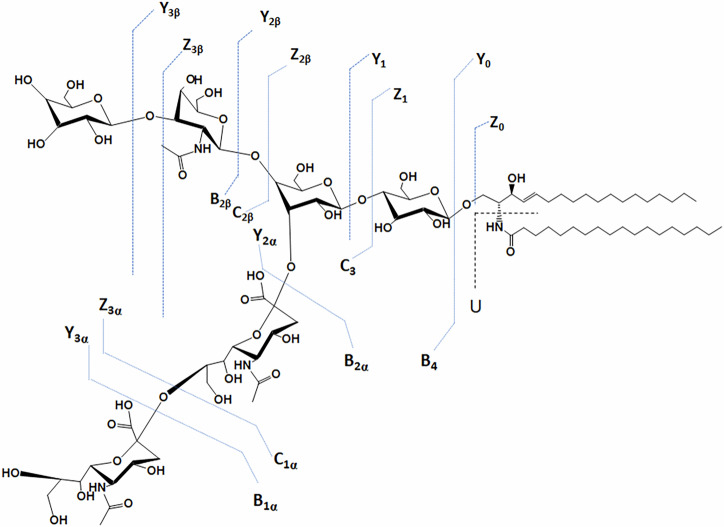
Fragmentation scheme of the glycan core for GD1(d18:1/18:0) species and fragmentation scheme of the ceramide. Y2β, Z3β and Y3β ions support the GD1b isomer while Y0, Z0, Z1 and U ions validate the composition of the ceramide.

In the case of TLR2D samples we have particularly focused the MS/MS analysis on the disialylated species, which present the highest expression in this mixture. The example given in Fig. 4 shows the detailed structural analysis by IMS MS/MS of the [M-2H^+^]^2-^ detected in TLR2D-M at *m/z* 917.471 which, according to mass calculation, corresponds to GD1 (d18:1/18:0). Obviously, CID MS/MS carried out at variable collision energy ramped from 30 to 60 eV gave rise to an elevated number of sequence ions, which occurred following the fragmentation of the glycosidic bonds and a few cross-ring cleavages. A list of the relevant fragment ions together with their assignment is provided in Supplementary Table 3. Additionally, a high intensity signal displaying a single mobility feature at 5.66 ms was identified and assigned, based on mass calculation, to doubly deprotonated GD1 (d18:1/18:0)(Fig. 4B). This species was subsequently isolated and subjected to CID MS/MS in the transfer cell. The main result of the fragmentation analysis shown in Fig. 4 is the generation of product ions from the non-reducing end, which confirms the positions of the two Neu5Ac residues along the oligosaccharide structure (Fig. 5 and Supplementary Table 3). The ion [M-H]^-^ at *m/z* 581.093, corresponding to B_2α_, serves as clear evidence for the presence of the disialo element (Neu5Ac)_2_.

The series of ions, comprising Y_2β_ detected as [M-2H]^2-^ at *m/z* 735.422, along with Z_3β_ ions at *m/z* 827.732 and Y_3β_ ions at *m/z* 836.829, unequivocally support the localization of both Neu5Ac residues at the inner galactose. This feature substantiates the presence of the disialo- element specifically at the internal Gal moiety, aligning with the structural configuration expected for the GD1b isomer (Fig. 5). The fragment ions Y_2β_/B_2α_ or B_4_/C_2β_ at *m/z* 888.532, C_3_/B_1β_ at *m/z* 963.391, Y_3β_/B_1α_ at *m/z* 1382.701, Z_3α_ at *m/z* 1526.922, and Y_3α_ at *m/z* 1544.891, all mono-deprotonated, collectively provide robust confirmation of the GD1 ganglioside structure. Each ion’s mass corresponds precisely to the theoretical expectations based on the GD1 proposed composition, validating specific glycosidic linkages and the arrangement of sialic acid residues within the ganglioside molecule. The presence of Y_2β_/B_2α_ or B_4_/C_2β_ at *m/z* 888.532 indicates characteristic glycan cleavage patterns, crucial for elucidating GD1 oligosaccharide structure and branching architecture. Similarly, C_3_/B_1β_ at *m/z* 963.391 confirms the location of sialic acid residues, essential for defining GD1 glycosidic sequence and overall molecular composition. Y_3β_/B_1α_ at *m/z* 1382.701 and Z_3α_ at *m/z* 1526.922 further complement these findings by highlighting additional glycan connectivity and residue positioning, underscoring the structural integrity of GD1. The fragment ion spectra confirm the composition (d18:1/18:0) of the aglycone, supported by the detection of mono-deprotonated Y_0_ at *m/z* 564.562, Z_0_ at *m/z* 546.547, and Z_1_ at *m/z* 708.618, indicative of the Glc-Cer structure. Additionally, U ion at *m/z* 282.287, related to the internal cleavage of the ceramide, further supports this composition.

### Lower amount of sterols indicates disrupted cholesterol synthesis in brain tissue of TLR2-deficient mice

**Fig. 6:**
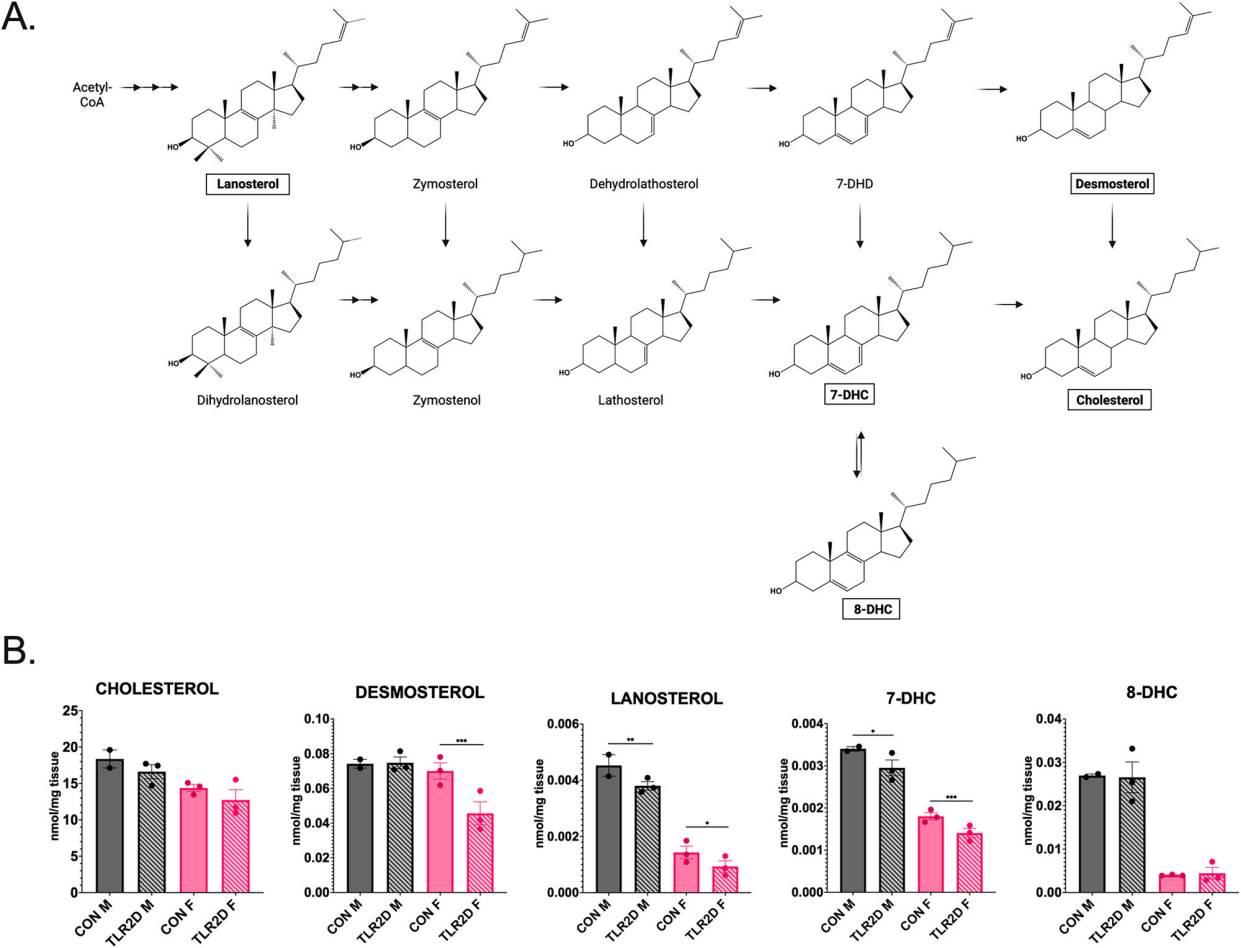
Intermediates in cholesterol metabolism extracted from cortical tissue samples of TLR2-deficient mice and control mice of both sexes. **A** Schematic representation of cholesterol metabolism, with the analyzed intermediates highlighted by rectangles; **B** Intermediates of cholesterol metabolism within organic phases upon extraction from brain tissue of TRL2-deficient and control mice, were quantified by sterol liquid chromatography-mass spectrometry with selected reaction monitoring (LC-MS/MS, SRM); quantities of cholesterol and distinct intermediates of its metabolism in TLR2-deficient male (TLR2D M) and female mice (TLR2D F) and matched controls (CON M; CON F). Data is shown as mean ± SEM; * = *p-*value ≤ 0.05, ** = *p-*value ≤ 0.01, *** = *p-*value ≤ 0.001; Unpaired two-tailed *t*-test; *n* = 3 animals per group.

We employed LC MS with selective reaction monitoring for detecting the presence and quantitative analysis of intermediates in cholesterol synthesis in organic phases extracted from cortical tissue derived from TLR2-deficient mice and sex/age-matched controls (Fig. 6). Specifically, examining the abundance of lanosterol, 7-dehydrocholesterol (7-DHC), 8-dehydrocholesterol (8-DHC), desmosterol and cholesterol unveiled two major distinctions between TLR2D and CON mice which imply the potential influence of TLR2 deficiency on cholesterol synthesis in brain tissue: (a) decreased average concentration of the analyzed sterols in TLR2D vs. CON; (b) sex-dependent pattern of sterol abundance, i.e., lower values of practically all sterols in CON females when compared with CON males, and more prominently decreased sterol abundance in TLR2D females than in TLR2D males (Fig. 6B).

Analysis of total cholesterol showed, in both female and male animals, approximately 10% lower concentration in cortical TLR2D *vs*. CON tissue; about 20% lower cholesterol concentration was found in CON-F than in CON-M and in TLR2D-F than in TLR2D-M (Fig. 6B). A sex-dependent trend in sterol quantification data was also observed when analyzing intermediates of cholesterol synthesis: the lowest concentrations of lanosterol, desmosterol, 7-DHC and 8-DHC were determined in TLR2D female mice, while lower concentrations of all sterols were characteristic for CON female in comparison with CON male mice (Fig. 6B). Significantly decreased sterol concentrations were assessed in cortical tissue of TLR2D mice when compared with control mice of both sexes, as follows: lanosterol (*p* = 0.016060, female mice; *p* = 0.004859, male mice; two-tailed unpaired *t*-tests; Fig. 6B); desmosterol (*p* = 0.000412, female mice; two-tailed unpaired *t*-tests; Fig. 6B); 7-DHC (*p* = 0.000961, female mice; *p* = 0.018928, male mice; two-tailed unpaired *t*-tests; Fig. 6B).

### TLR2 deficiency is associated with submembrane redistribution of gangliosides and cholesterol

**Fig. 7:**
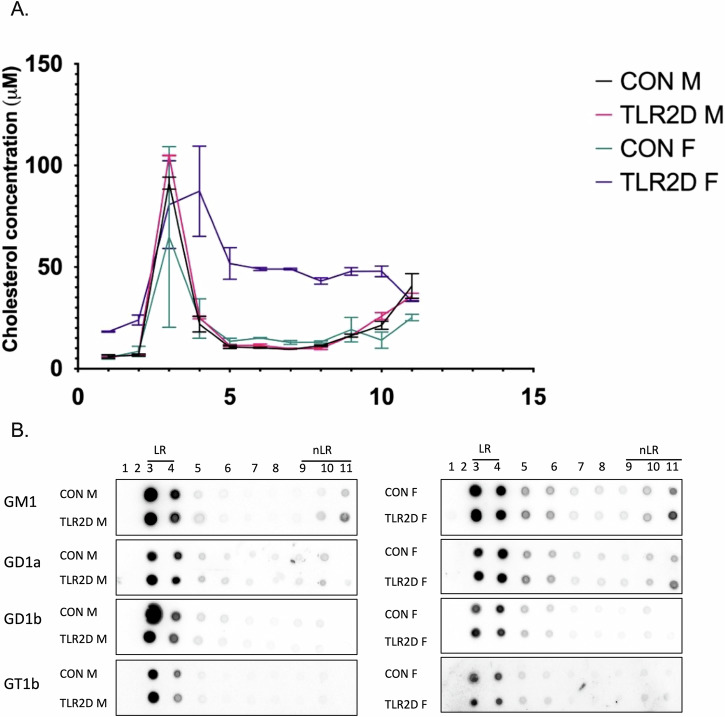
Disturbances in submembrane localization of cholesterol and specific ganglioside species related to TLR2 deficiency. **A** Distribution of cholesterol within lipid raft (LR) and non-lipid raft (nLR) fractions among TLR2-deficient male (TLR2D M) and female mice (TLR2D F) and matched controls (CON M, CON F). Cholesterol concentration was determined in each of 10 membrane fractions upon membrane sub-fractionation. Data is shown as mean ± SEM, *n* = 3 animals per group; **B** Representative dot-blots of 4 major brain ganglioside species in membrane fractions upon membrane sub-fractionation for each analyzed group (*n* = 3 animals per group).

To delve into the likely effects of TLR2 deficiency on the composition and rearrangements of lipids within membrane domains, we analyzed the abundance of cholesterol and gangliosides in LR and non-LR membrane fractions isolated from cortical tissue samples derived from TLR2D and control mice. After confirmation of the successful membrane sub-fractionation by determining the adequate distribution of LR and non-LR protein markers (Supplementary Fig. 5), membrane fractions were further utilized for quantification of cholesterol and evaluation of submembrane localization of gangliosides. Interestingly, sex differences in cholesterol content were confirmed even at the submembrane level: the highest cholesterol concentration was found in LR fractions in CON and TLR2D male mice while distribution of cholesterol seemed to be much more dispersed in both CON and TLR2D female mice (Fig. 7A). The presence of four major brain ganglioside species (GT1b, GD1b, GD1a and GM1) in membrane fractions was determined by dot-blot analysis which showed a “leakage” of gangliosides from LRs to nonLRs, particularly of GM1 and GD1a in TLR2D female mice (Fig. 7B, Supplementary Fig. 6). Briefly, these findings nicely uphold the presumed association of TLR2 deficiency with rearrangements of membrane lipids, specifically perceived as the reshuffling of cholesterol and gangliosides from LRs, their expected major sites of action, to non-LRs.

### RNA sequencing reveals association of TLR2 deficiency with sex-dependent differences in transcriptomic profiles

**Fig. 8:**
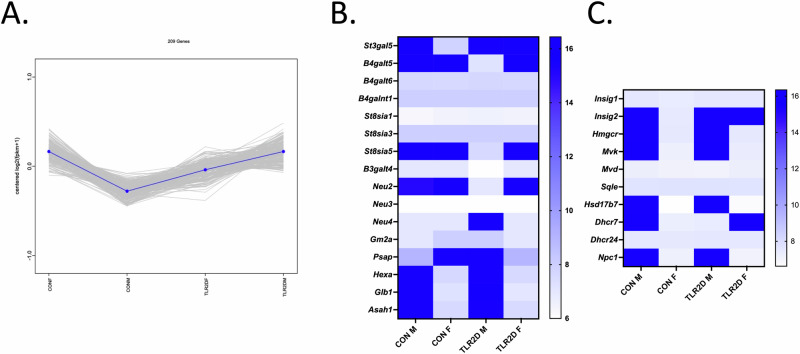
RNA sequencing analysis of brain tissue derived from TLR2 deficient and control mice of both sexes. **A** Overall co-expression analysis of 209 differentially expressed genes (DEGs) across four experimental groups: CON F (control female), CON M (control male), TLR2D F (TLR2-deficient female), and TLR2D M (TLR2-deficient male). Gray lines represent centered log₂(FPKM + 1) expression levels of individual genes; the blue line shows the average expression trend. Heatmaps show log₁₀-transformed FPM values for genes involved in ganglioside (**B**) and cholesterol (**C**) metabolism. Rows represent genes, columns represent groups, with blue indicating higher and white lower expression. Gene annotations and protein product information for genes shown in (**B**, **C**) are given in Supplementary Table 5.

Evident sex-dependent transcriptomic differences were demonstrated by means of RNAseq analysis both within groups of control and TLR2D mice (Supplementary Table 4). The highest number of differentially expressed genes (DEGs) was observed between control males and females, totaling 574 genes (170 upregulated, 404 downregulated). A total of 314 genes exhibited differential expression in TLR2D males and females, with 181 downregulated and 133 upregulated genes. When comparing TLR2D females to control females, 356 DEGs were identified, of which 103 were upregulated and 253 downregulated. Additionally, 565 DEGs were found when comparing control males to TLR2D males, with 275 upregulated and 290 downregulated genes. To investigate how TLR2 impairment and sex differences affect coordinated transcriptional changes, we conducted a co-expression analysis on the differentially expressed genes. This analysis identified 209 genes with similar expression patterns across all groups, visualized as centered log2(FPKM + 1) profiles (Fig. 8A). The results revealed a clear pattern: control males displayed lower gene expression levels than control females, TLR2D females had intermediate levels, and TLR2D males showed the highest expression. Further analysis of transcriptomic data focused on expression patterns of genes involved in ganglioside and sterol metabolism across all animal groups (Supplementary Table 5). Log10-transformed FPM data showed distinct expression patterns, with several genes exhibiting reduced expression in TLR2D mice compared to controls. Notably, sex-based differences were observed within both the control and TLR2D groups (Fig. 8B). For instance, lower expression of *B4galt5* and *St8sia5* coding for enzymes involved in ganglioside synthesis, and of *Neu2* involved in cytosolic degradation of gangliosides, was found in TLR2D males in comparison to CON M, CON F and TLR2D F (Fig. 8B). In CON F, expression of *St3gal5* coding for GM3 synthase was lower than in TLR2D females and both control and TLR2D male animals (Fig. 8B). On the other hand, gene expression of the catabolic machinery (*Hexa*, *Glb1*, *Asah1*) was higher in both control and TLR2D M when compared with CON F and TLR2D F mice, with exception of *Neu4* responsible for lysosomal degradation of glycolipids and glycoproteins found with highest expression in TLR2D F mice (Fig. 8B). Log10-transformed FPM values for genes related to cholesterol metabolism also varied among animal groups, and indicated even more striking sex-related differences seen as lower expression of majority of cholesterogenic genes in female vs male mice (Fig. 2C). In the case of *Insig2* and *Dhcr7*, higher expression was detected in TLR2D female vs control female mice, reaching the gene expression level observed in males; also, in TLR2D male mice, the *Dhcr7* expression was lower than in control male animals (Fig. 8C).

## Discussion

Results of this study confirm the hypothesized impact of TLR2 deficiency on the structural and compositional rearrangements of membrane lipids in the brain, revealed by in-depth lipidomic analysis of gangliosides and sterols isolated from cortical tissue samples of TLR2-deficient and control mice. Moreover, expression patterns of brain gangliosides and sterols turned out to be sex-specific, both in TLR2D and control mice. The main findings of the lipidomic profiling, as summarized in Table 2, along with corroborating data obtained by RNA sequencing (Fig. 8), clearly point to the relationship of TLR2, gangliosides and cholesterol in murine brain tissue.

**Table 2:** Brain gangliosides and sterols in male and female mice with TLR2-deficiency in comparison with control mice – a summarized overview

	Glycolipidomic analysis	Sterol analysis
BRAIN TISSUE	Lower total amount of brain gangliosides in TLR2D than in CON mice; both TLR2D F and CON F express lower concentration of total gangliosides when compared with TLR2D and CON male mice (Supplementary Fig. 2.) ESI-MS analysis shows overall and sex-dependent greater abundance, structural diversity, glycan and ceramide moiety heterogeneity, and presence of modifications of ganglioside species in TLR2D than in CON mice (Supplementary Tables 1. and 2.; Figs. 1, 2 and 3) ESI-MS analysis indicates sex-related differences in brain ganglioside profiles: disialo-species (GD1) predominate in male mice, polysialo-species (GT1, GQ1) more abundant in female mice; fucosylated species more common in males, *O*-acetylated ones in females; higher presence of GM3, GM2, GD2 in TLR2D than in CON mice, the difference being more prominent in female mice; several ganglioside species and modifications identified as unique markers of TLR2D vs CON and/or F vs M samples (Table 2., Supplementary Tables 1. and 2, Fig. 3.) IMS CID MS/MS resolves the structure of GD1b isomer specifically expressed in TLR2D-M (Fig. 5.)	LC MS determines lower total concentration of cholesterol in TLR2D than in CON mice, decrease being more prominent in TRL2D F mice; (Fig. 6) LC MS analysis shows significantly decreased concentration of all analysed intermediates in cholesterol synthesis in TLR2D vs CON, most prominent decrease detected in TRL2D female mice (Fig. 6)
MEMBRANE FRACTIONS	Dot-blot analysis shows presence of GT1b, GD1b, GD1a and GM1 in membrane fractions isolated from brain tissue samples; redistribution of gangliosides within rafts and non-rafts was determined, noticeable particularly for GM1 and GD1a in TLR2D female mice (Fig. 7B)	Quantification of total cholesterol in isolated membrane fractions shows higher cholesterol concentration in lipid rafts of CON and TLR2D male mice than in female counterparts (Fig. 7A). Distribution of cholesterol within membrane fractions is more dispersed in both CON and TLR2D female mice than in male counterparts (Fig. 7A)

Regarding compositional and structural analysis of brain gangliosides, utilization of highly-sensitive mass spectrometry techniques uncovered a probable adaptation of glycosphingolipid synthesis machinery occurring due to the lack of transmembrane protein TLR2. This may explain detection of individual ganglioside species characterized by greater structural diversity, heterogeneity of both glycan and ceramide moiety and presence of modifications such as acetylations, *O*-acetylations, and fucosylations in TLR2D mice (Fig. 3, Tables 2 and 3). Another intriguing observation are sex-dependent differences in the brain ganglioside profiles when comparing both control male and female mice and TLR2-deficient male and female mice (Fig. 3, Table 2). Supportive evidence provided by preliminary RNA sequencing analysis, showing different transcriptome profiles of genes involved in ganglioside metabolism, may be linked to detected distinct and sex-specific brain gangliosidome features in TLR2D vs controls (Fig. 8B, Supplementary Tables 4 and 5). When analyzing ganglioside structural modifications, we found that fucosylations are more common in both control and TLR2D males than in female mice, and identified shared species (Fuc-GM1 in control males and females; Fuc-GD1 in TRL2D males and females) suggesting that certain fucosylated gangliosides are essential and conserved due to their biological requirement or house-keeping role in cellular functions (Table 1)^40–42^. Furthermore, for acetylations we detected: a higher number of *O*-acetylated species in TLR2D compared to control samples in males (Table 2); higher structural diversity of *O*-Ac species in TLR2D males than in any other analyzed group (Table 1); acetylated gangliosides, such as (CH_3_COO^-^)_2_GD1(d18:1/18:0) (Supplementary Tables 1 and 2). In general, changes in glycosphingolipid synthesis and various structural modifications with consequent altered glycolipid composition of membranes could indicate adaptive responses to the absence of specific gene(s), or physico-chemical adjustment of membrane lipid constituents to compensate for the lack of proteins in the lipid raft functional cluster^43–46^. Whether these modifications and structural membrane adaptations have functional consequences for TLR2, whose signaling actions are influenced by lipid microdomain environment, should be further explored^47^.

Another important finding of the structural characterization of ganglioside species in TLR2-deficiency refers to higher occurrence of simple gangliosides compared to controls, and significant sex-specific differences in ganglioside compositional and structural features in both control and TRL2-deficient mice (Supplementary Tables 1 and 2, Fig. 3). Data on sex-differences related to the composition and abundance of brain gangliosides are rather scarce, however, several studies report age-dependent changes in ganglioside content in male and female rat brains, as well as sex-specific differences in human brain ganglioside composition in Parkinson’s disease^48,49^. That being said, our study, which utilized a powerful and sensitive MS approaches, firmly demonstrates an unambiguous sex-related distinction in abundance and structural diversity of brain ganglioside species in both control and TLR2D mice. Of note are corroborating results of transcriptomic analysis, showing sex-dependent hierarchical clustering of DEGs and expression of genes involved in synthesis and degradation of gangliosides (Fig. 8, Supplementary Tables 4 and 5). In addition, functional annotation analysis derived from RNAseq indicates that the highest number of DEGs is linked to neuroactive ligand-receptor interactions, cancer, and calcium signaling pathways, the processes with documented roles of both gangliosides and TLR2.

This study also dealt with determining the amounts of cholesterol and intermediates of cholesterol synthesis in cortical brain tissue samples by LC-MS, and showed that the total concentrations of cholesterol, as well as nearly all analyzed sterols, were lower in TLR2-deficient male and female mice when compared to controls (Fig. 6). The lowest sterol concentrations were consistently found in TLR2D females; decreased sterol amounts were also determined in female controls in comparison to male controls (Fig. 6). The observation on sex-differences in cholesterol metabolism is not quite new, particularly regarding cholesterol concentration in serum, but only few papers report on sex-different expression of regulatory cholesterogenic enzymes in mammalian brain tissue^50–52^. Also, there is evidence on sex-specific alteration of brain lipids profiles, including gangliosides, in mouse model lacking *Abca7* (gene responsible for membrane transport of cholesterol)^53^. Thus the results of the RNAseq analysis performed in this study, bring additional notable finding by assessing lower expression of cholesterogenic genes, including the regulatory *Hmgcr*, in cortical tissue of both control and TLR2D females when compared to male mice (Fig. 8C). Interestingly, reduced level of circulating corticosterone is one of the phenotypic traits of TLR2-deficient mice, that may be linked to the observed reduced availability of cholesterol, a precursor of steroid synthesis^54^.

Moreover, the sex-specific lipidomic profiles determined in cortical tissue were in part reproducible at the membrane level. Namely, this study tracked the redistribution of gangliosides and cholesterol within isolated membrane fractions, finding a pronounced reshuffling of cholesterol to non-rafts in TLR2D female mice (Fig. 7). A similar disturbed sub-membrane localization of gangliosides, translocating from rafts in favor of non-rafts, was recognized particularly for GM1 and GD1a in TRL2D female mice (Fig. 7, Supplementary Fig. 6). Another important lipid resident of LRs, sphingomyelin, forms intermolecular interactions with both cholesterol and gangliosides and contributes to dynamic properties of membrane architecture^55^. This work focused to detailed lipidomic analysis of brain gangliosides and cholesterol in TLR2 deficiency, however joint actions of sphingomyelin, gangliosides and cholesterol in modulation of TLR2 signaling deserve further exploration, as similar effects have been described for TLR4 recruitment to LRs^47^.

Based on the results of our study and supportive literature evidence for structural and functional links between TLR2, gangliosides and cholesterol, involvement of TLR2 in innate immunity, neurodevelopment and several neuropsychiatric disorders, we propose that their roles in brain tissue converge with molecular mechanisms of processes related to neuroimmunity. Indeed, evidence points to shared roles of TLR2, gangliosides and cholesterol in disorders characterized by inflammation, such as insulin resistance, cancer and neurodegeneration; some of these insights are derived from research done in TLR2-deficient mice whose phenotype includes altered energy metabolism, increased susceptibility to neoplasms, unregulated tissue responses in brain ischemia, neurobehavioral dysfunctions^56–60^. In addition, the results of our study pointing to sex-specific differences in brain glycolipidome and sterolome in TLR2 deficiency are well in line with reported metabolic sex dimorphism in brain, particularly referring to sex bias of immune cells^61,62^.

To conclude, shown results provide evidence on the sex-specific structural and functional relationship between TLR2, gangliosides and cholesterol, underlying membrane reorganization driven by TRL2-deficiency. The study presents detailed lipidomic data and elaborate structural characterization of gangliosides and sterols isolated from brain tissue derived from TLR2-deficient and control mice of both sexes, with complementary information provided by preliminary transcriptomic analysis. It identifies ganglioside structures that serve as unique markers for TLR2-deficiency in males and females, detects probable decreases in cholesterol synthesis in TLR2-deficiency with implications for neuro-steroids actions, and demonstrates sex-specific changes in ganglioside and sterol composition accompanying TLR2-deficiency in brain tissue samples and within membrane domains. Although this study primarily focuses on structural characterization of gangliosides and sterols, its implications highlight the need for further research into the role of intricate functional networking of membrane constituents in complex brain processes. Our findings advocate for a broader perspective on the TLR2-gangliosides-cholesterol axis which my act as a relay point integrating environmental stimuli and regulating neuroimmune response in a sex-dependent manner.

## Materials and methods

### Chemicals and reagents

Isoflurane was purchased from Vetpharma Animal Health S.L., Barcelona, Spain. Usual laboratory buffer reagents, sucrose and organic solvents were purchased from Kemika d.d., Zagreb, Croatia. HPLC grade solvents for ganglioside extraction, purification, HPTLC and MS analyses were purchased from Merck, Darmstadt, Germany. Sephadex G25 gel, protease inhibitor cocktail, Brij O20, bovine serum albumin (BSA), HPTLC silica gel 60 glass plates, resorcinol and *N*-acetylneuraminic standard were purchased from Sigma-Aldrich (St. Louis, MO, USA). Solvents and chemicals for sterol analysis were purchased from Sigma-Aldrich Co (St. Louis, MO). HPLC grade solvents were purchased from Thermo Fisher Scientific Inc. (Waltham, MA). Isotopically labeled sterol standards used in this study are available from Kerafast, Inc. (Boston, MA). Amplex Red Cholesterol Assay Kit and Western blot reagents NuPage LDS sample buffer, NuPage antioxidant, NuPage 4–12% precast Bis-Tris gels, NuPAGE MOPS SDS Running Buffer, NuPAGE Transfer Buffer, prestained protein ladder and Supersignal West FEMTO maximum Sensitivity Substrate for detecting chemiluminescence were all purchased from Thermo Fisher Scientific, Life Technologies Corporation, Carlsbad, CA, USA. PVDF blotting membranes were purchased from Macherey-Nagel, Düren Germany and nitrocellulose blotting membranes were purchased from Bio-Rad, Hercules, CA, USA. Non-fat dry milk used for blocking in Western blotting and dot blotting was from Santa Cruz Biotechnology, Dallas, TX, USA. The preparation of monoclonal anti-ganglioside antibodies is described previously^63^; list and the corresponding details for all primary and secondary antibodies used for Western blotting and dot blotting are given in Table 3.

**Table 3:** List of primary and secondary antibodies used for Western and dot blotting

Antibody	Host species	Supplier	Cat. number	Dilution
Primary antibodies				
Anti-transferrin receptor	Mouse	Thermo Fisher, Life Technologies Corporation, Carlsbad, CA, USA	136800	1:2000
Anti-Thy1/CD90	Rabbit	Abcam, Cambridge, UK	307736	1:1000
Anti-GM1: horseradish peroxidase (HRP)-conjugated CTB (cholera toxin subunit B)		Thermo Fisher, Life Technologies Corporation, Carlsbad, CA, USA	C34780	1:50,000
Anti-GD1a	Mouse	Monoclonal antibodies prepared and validated as reported^63^		0.64 μg/mL
Anti-GD1b				2 μg/mL
Anti-GT1b				1.84 μg/mL
Secondary antibodies				
Anti-mouse HRP	Donkey	Jackson ImmunoResearch Europe Ltd., Ely, UK		715-035-150
Anti-rabbit HRP	Donkey			711-035-152

### Animals

A mouse model with genetically modified expression of TLR2, B6.129-Tlr2^tm1Kir/J^ was backcrossed across more than 20 generations to B6-Tyr^c-Brd^*/*BrdCrCrl resulting in B6-Tlr2^tm1Kir /Gaj^ transgenic line on an albino background (TLR2-deficient, denoted in the manuscript as TLR2D). TLR2D mice used in this study were compared to sex- and age-matched wild-type littermates (controls, denoted in the manuscript as CON). A total of 45 young adult mice, 3 months old, were used for this study. The genotype of all animals was confirmed using the official protocol (number 26453, version 4.2) available on the Jackson Laboratories (The Jackson Laboratories, Bar Harbor, USA) website. The animals were housed in groups under standardized conditions, including controlled temperature and humidity, a 12 h light/dark cycle, and unrestricted access to food and water. For all experiments, the animals were euthanized via an isoflurane overdose, followed by decapitation. The brains were carefully extracted and the cortex was dissected using neuroanatomical landmarks. Tissue samples were snap frozen and kept at −80 °C until further use. All experimental procedures were performed in accordance with the ARRIVE guidelines and approved by regional ethics committees for scientific experiments and by the appropriate institutions in agreement with institutional and government guidelines. The overview of experimental design is presented in Supplementary Fig. 1.

### Lipid extraction and purification

Ganglioside extraction and purification was performed in accordance with following protocols: cortices were homogenized in ice-cold distilled water (W) in a Potter–Elvehjem glass-Teflon homogenizer by 15 strokes; lipids were extracted using organic solvents chloroform (C): methanol (M) (1:2, by vol.), followed by phase partition and repartition by adding M and W to a final volume ratio 1:1:0.7; upper phases were collected, evaporated to dryness and further purified by gel filtration on Sephadex-G25 columns; dried purified samples were stored at −20 °C until analysis; lower organic phases following phase partition and repartition were evaporated to dryness and stored at −20 °C until use for sterol analysis (Supplementary Fig. 1)^25,64^.

### Ganglioside quantification and high-performance thin layer chromatography

Quantitative analysis of ganglioside-bound sialic acid content was determined by spectrophotometry^64,65^. The absorbance values of samples and *N*-acetylneuraminic acid used as a standard in a range of known concentrations were determined at 580 nm. The contents of ganglioside-bound sialic acids are expressed as microgram of ganglioside-bound sialic acids per gram of fresh tissue w.w. The samples were further qualitatively analyzed by HPTLC^41^. Purified samples were dissolved in C:M:W (60:30:4.5, by vol.), and the aliquots were spotted on to the HPTLC plate, resolved and detected by resorcinol-HCl reagent^25,64^. Densitometry was performed on a ChemiDoc MP imager (Bio-Rad, Hercules, CA, USA) using Image Lab software (Bio-Rad, Hercules, CA, USA) and bands quantified using ImageJ analysis software (NIH, Bethesda, MD, USA) as percentage in immunoreactivity intensity. Relative quantification of individual ganglioside species is expressed as their relative proportions (%) in total ganglioside content in the analyzed sample.

### Ion mobility and collision-induced dissociation mass spectrometry (IMS MS and CID MS/MS)

All IMS MS experiments performed in this study were conducted on a Synapt G2S mass spectrometer (Waters, Milford, MA, USA) equipped with a nanoESI source. The screening and tandem mass spectra were acquired in negative ion mode under identical conditions for all samples, in the mass range of 400–2500 m/z, with a scan speed of 1 scan/s. The minimum S/N (signal-to-noise) value for ion identification was 70 counts/s. Stock solutions were prepared by dissolving the dried ganglioside extracts in HPLC grade methanol and stored at −27 °C until the IMS MS analysis. Aliquots of the stock solutions were diluted in methanol to yield the working samples at a concentration of approximately 5 pmol μL^-1^, calculated for an average relative molecular mass of 2000. To perform nanoESI IMS MS screening, a 10 μL from each of the 5 pmol μL^−1^ methanolic solutions of gangliosides isolated from cortical tissue homogenates were infused one after the other and under identical conditions into the Synapt G2S instrument operating in the negative ion mode. For every measurement the signal was acquired for 4 min, during which a two-dimensional data set was generated revealing significant reduction in spectral noise and effective separation of components into mobility families across various drift times. The 2D plot highlighted the separation of gangliosides (GGs) based on charge state, carbohydrate chain length, and degree of sialylation, facilitating the detection of numerous species, including low-abundance variants with biological importance. In all cases, the signal was acquired for 2 min in negative ion mode. The samples were loaded into a 10 cm long nanoESI emitter with the following parameters: ID 1.2 mm, OD 1.5 mm, tip size 10 µm, taper length 4 mm. The ESI voltage was applied to a 0.25 mm platinum wire inserted into the solution. The potential values applied to the wire and the cone were adjusted to achieve efficient ionization of the molecules and minimal in-source fragmentation. A continuous spray was initiated and maintained at 1.3 kV ESI and 40 V cone potentials. For efficient desolvation and separation of ganglioside ions, the other MS and IMS parameters were set as follows: source block temperature 100 °C; desolvation gas flow rate 100 L h^−1^; IMS gas flow 90 mL min^−1^; IMS wave velocity 650 m s^−1^; IMS wave height 40 V; TOF analyzer operated in V-mode with an average mass resolution of 20,000. Tandem MS experiments were performed by CID in the transfer cell, with the low mass (LM) and high mass (HM) resolution parameters set at 13 and 15, respectively. To generate a high coverage of sequence ions with an elevated number of diagnostic ions, the MS/MS scans were acquired using variable collision energies within the range of 30–70 eV, laboratory values (E_lab).

### Ganglioside abbreviation and assignment of the mass spectra

For ganglioside assignment, the abbreviation system introduced by Svennerholm together with the recommendations of IUPAC-IUB Commission on Biochemical Nomenclature was applied^64,65^:

GM1 - II^3^-α-Neu5Ac-Gg_4_Cer; GM2 - II^3^-α-Neu5Ac-Gg_3_Cer; GM3 - II^3^-α-Neu5Ac-LacCer; GD1 - II^3^-α-(Neu5Ac)_2_-Gg_4_Cer; GD2 - II^3^-α-(Neu5Ac)_2_-Gg_3_Cer; GD3 - II^3^-α-(Neu5Ac)_2_-LacCer; GT1 - II^3^-α-(Neu5Ac)_3_-Gg_4_Cer; GT2 - II^3^-α-(Neu5Ac)_3_-Gg_3_Cer; GT3 - II^3^-α-(Neu5Ac)_3_-LacCer; GQ1 - II^3^-α-(Neu5Ac)_4_-Gg_4_Cer; GQ2 - II^3^-α-(Neu5Ac)_4_-Gg_3_Cer; GP1 - II^3^-α-(Neu5Ac)_5_-Gg4Cer; GP2 - II^3^-α-(Neu5Ac)_5_-Gg_3_Cer; GH1 - II^3^-α-(Neu5Ac)_6_-Gg_4_Cer; GH2 - II^3^-α-(Neu5Ac)_6_-Gg_3_Cer; GS1 - II^3^-α-(Neu5Ac)_7_-Gg_4_Cer; GS2 - II^3^-α-(Neu5Ac)_7_-Gg_3_Cer; GO1 - II^3^-α-(Neu5Ac)_8_-Gg_4_Cer. The spectral signals were attributed to ganglioside species by exact mass calculations, considering the monoisotopic masses and the known data on these substrates and their biosynthesis criteria^66–68^. The carbohydrate sequence ions generated by CID MS/MS fragmentation experiments were assigned according to the nomenclature introduced by Domon and Costello^69^, revised by Costello et al.^70^. The ceramide fragment ions were assigned according to the nomenclature established by Ann and Adams^71^.

### Sterol liquid-chromatography mass spectrometry with selected reaction monitoring analyses

Samples were processed according to optimized protocols^72,73^. Dried lower organic phase was used for 4-Phenyl-1,2,4-triazole-3,5-dione (PTAD) derivatization^74,75^. The derivatization reagent PTAD dissolved in M was added to dried samples and the reaction was fully completed after 30 min at ambient temperature. The samples were placed in an Acquity UPLC system equipped with an ANSI-compliant well plate holder coupled to a Thermo Scientific TSQ Quantis mass spectrometer equipped with an APCI source (Thermo Fisher, Life Technologies Corporation, Carlsbad, CA, USA). Then 10 μL was injected onto the column (Phenomenex Luna Omega C18, 1.6 μm, 100 Å, 2.1 mm × 100 mm) with 90% M and 10% ACN (0.1% v/v acetic acid) mobile phase for 1.7 min runtime at a flow rate of 500 μL/min. Natural sterols were analyzed by selective reaction monitoring (SRM) using the following transitions: Cholesterol 369 → 369, 7-dehydrocholesterol (7-DHC) 560 → 365, desmosterol 592 → 560, lanosterol 634 → 602, with retention times of 0.7, 0.4, 0.3 and 0.3 min, respectively. SRMs for the internal standards were set to: *d*7-Chol 376 → 376, 8-DHC 558 → 363, *d*7-8-DHC 565 → 370, d7-7-DHC 567 → 372, 13C3-desmosterol 595 → 563, 13C3-lanosterol 637 → 605^76^.

### Lipid raft isolation

Lipid rafts were isolated by discontinuous sucrose gradients ultracentrifugation^77^. Briefly, 70 ± 5 mg of mouse cortices were homogenized, the nuclear fraction removed, and the cell membrane pellet obtained by centrifugation (100,000 x g at +4 °C, 30 min; ultracentrifuge Beckman Optima XL-80 K, Beckman Coulter, Brea, CA, USA). The pellet was homogenized in a buffer containing Brij O20 and ultracentrifuged (140,000 x g, at +4 °C, 18 h) in a discontinuous sucrose gradient (85% sucrose mixed with the sample in 1:1 ratio, overlaid with 35% sucrose solution and 3% sucrose solution. After centrifugation, fractions were collected from top to bottom and analyzed by Western blotting to assess the lipid raft (LR) and the bulk membrane (non-lipid raft; nLR) distribution of proteins of interest. Transferrin receptor (TfR) is considered an nLR marker and Thy1 is a LR marker^78,79^.

### Western blotting

Western blotting was performed utilizing described procedures^25,77^. Equal volumes of isolated lipid raft fractions (*n* = 11/sample) were loaded onto precast 4–12% Bis-Tris gels to directly compare protein distribution across fractions^78^ (Supplementary Fig. 5). The gels were resolved in MOPS running buffer, transferred to PVDF membrane, incubated with blocking reagent and primary antibodies overnight at 4 °C, followed by the appropriate secondary antibodies. The antibody details are given in Table 3. Protein bands were visualized using chemiluminescence according to the manufacturer’s instructions and the visualization was performed on the ChemiDoc MP Imaging System (Bio-Rad, Hercules, CA, USA).

### Dot blotting

A 1 μL aliquots of each fraction obtained by lipid raft isolation (*n* = 10/sample) were spotted onto the nitrocellulose membrane and left to dry before being blocked in 5% non-fat dry milk in PBST for 1 h at ambient temperature. Blots were incubated overnight at 4 °C in anti-ganglioside primary antibody solutions prepared in blocking solution, followed by the incubation in appropriate secondary antibodies diluted in PBST for 1 h at ambient temperature. The antibody details are given in Table 3. Visualization was performed as described for Western blotting.

### Determination of cholesterol concentration in isolated membrane fractions

Cholesterol concentration in each fraction after lipid raft isolation (10 fractions per tissue sample; *n* = 3 animals per group) was performed using the ready-made Amplex Red Cholesterol Assay Kit according to the manufacturer’s instructions. The fluorescence was measured at the GloMax Discover plate reader (Promega, Madison, WI, USA).

### RNA isolation and transcriptome sequencing

Total RNA was extracted from the cerebrocortical tissue of three control and three TLR2D mice of both sexes using TRIzol Reagent (Life Technologies, CA, USA). RNA concentration and purity were measured using a NanoDrop 2000 spectrophotometer (Thermo Fisher Scientific, DE, USA), and integrity was assessed with the RNA Nano 6000 Assay Kit on an Agilent Bioanalyzer 2100 (Agilent Technologies, CA, USA). 1 μg of total RNA from each sample was used to prepare libraries with the NEBNext Ultra RNA Library Prep Kit for Illumina (NEB, USA). mRNA was isolated using Poly-T oligo-attached magnetic beads, fragmented into fragments of approximately 240 bp using the AMPure XP system (Beckman Coulter, USA), and then reverse-transcribed into cDNA with random hexamer priming using Phusion High-Fidelity DNA Polymerase. The cDNA was subsequently purified and quality assessed using the Bioanalyzer 2100. Using the TruSeq PE Cluster Kit v4-cBot-HS (Illumina), clusters were created using a cBot system, and paired-end sequencing was carried out on an Illumina platform. Adapters, poly-N sequences, and low-quality reads were eliminated from the raw data using filtering. Clean readings were assessed for duplication levels, GC content, Q20, and Q30. Fragments per kilobase per million mapped reads (FPKM) were used to quantify gene expression. DESeq2 was used to evaluate differential expression, and genes with adjusted *p *< 0.05 were considered differentially expressed using the Benjamini-Hochberg technique. KOBAS was used for KEGG pathway enrichment, and GOseq was used for GO enrichment analysis of differentially expressed genes (DEGs), all analyses being performed on the BMKCloud platform (www.biocloud.net).

### Statistical analysis and data processing

All graphics and statistical analyses were conducted using GraphPad Prism (GraphPad Software, Boston, MA, USA). The data in the graphs is presented as mean ± SEM. For IMS MS data acquisition and processing, MassLynx (version V4.1, SCN 855) and Waters Driftscope (version V2.7) software were employed. An unpaired, two-tailed t-test was conducted for sterol analysis. In all instances, a *p*-value below 0.05 indicated statistically significant results.

### Ethics statement

Experimental procedures were performed in accordance with the ARRIVE guidelines and the project IP-2016-06-8636 to S.K.-B. approved by regional ethics committees and institutional ethical board (approval issued by School of Medicine, Zagreb University, No: 380-59-10106-17-100/134).

### Reporting summary

Further information on research design is available in the Nature Portfolio Reporting Summary linked to this article.

## Supplementary information

Supplementary material

Reporting summary

## Data Availability

The access to numerical source data for graphs and charts, along with other raw data will be available upon reasonable requests from corresponding authors.
