# Supplementary material for: Deficiency of Toll-like receptor 2 is a driver of sex-related compositional and structural rearrangements of membrane lipids
Source: Commun Chem. 2025 Dec 6;8:395. doi: 10.1038/s42004-025-01766-x (PMC12706035; doi:10.1038/s42004-025-01766-x)
Supplement: Supplementary file 1 — Supplementary material [file 42004_2025_1766_MOESM1_ESM.pdf]

SUPPLEMENTARY MATERIAL

I. SUPPLEMENTARY FIGURES

Supplementary Figure 1. Experimental design of the study

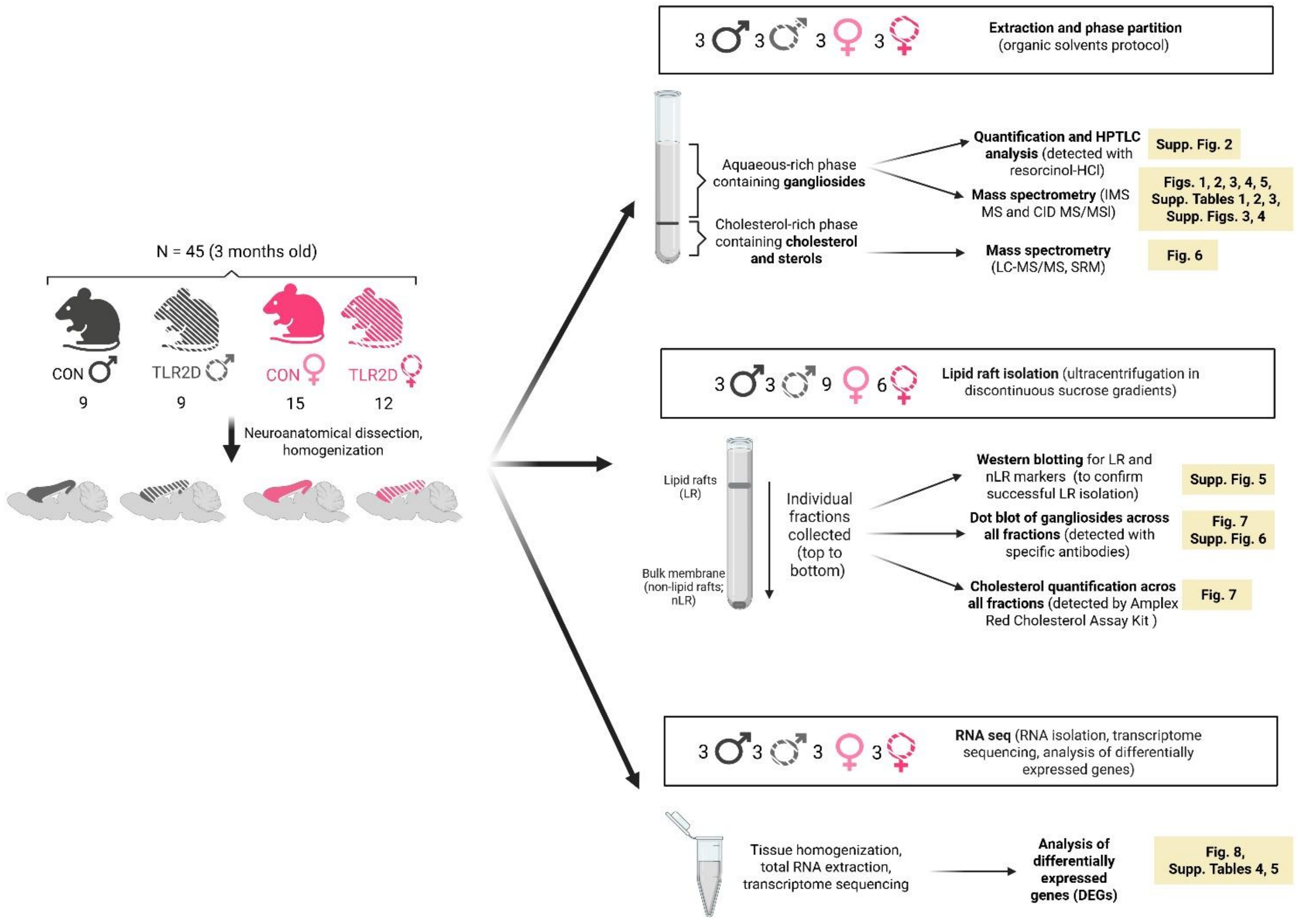

Supplementary Figure 2. Ganglioside analysis and quantification by high-performance thin layer chromatography in TLR2-deficient male and female mice and matched controls

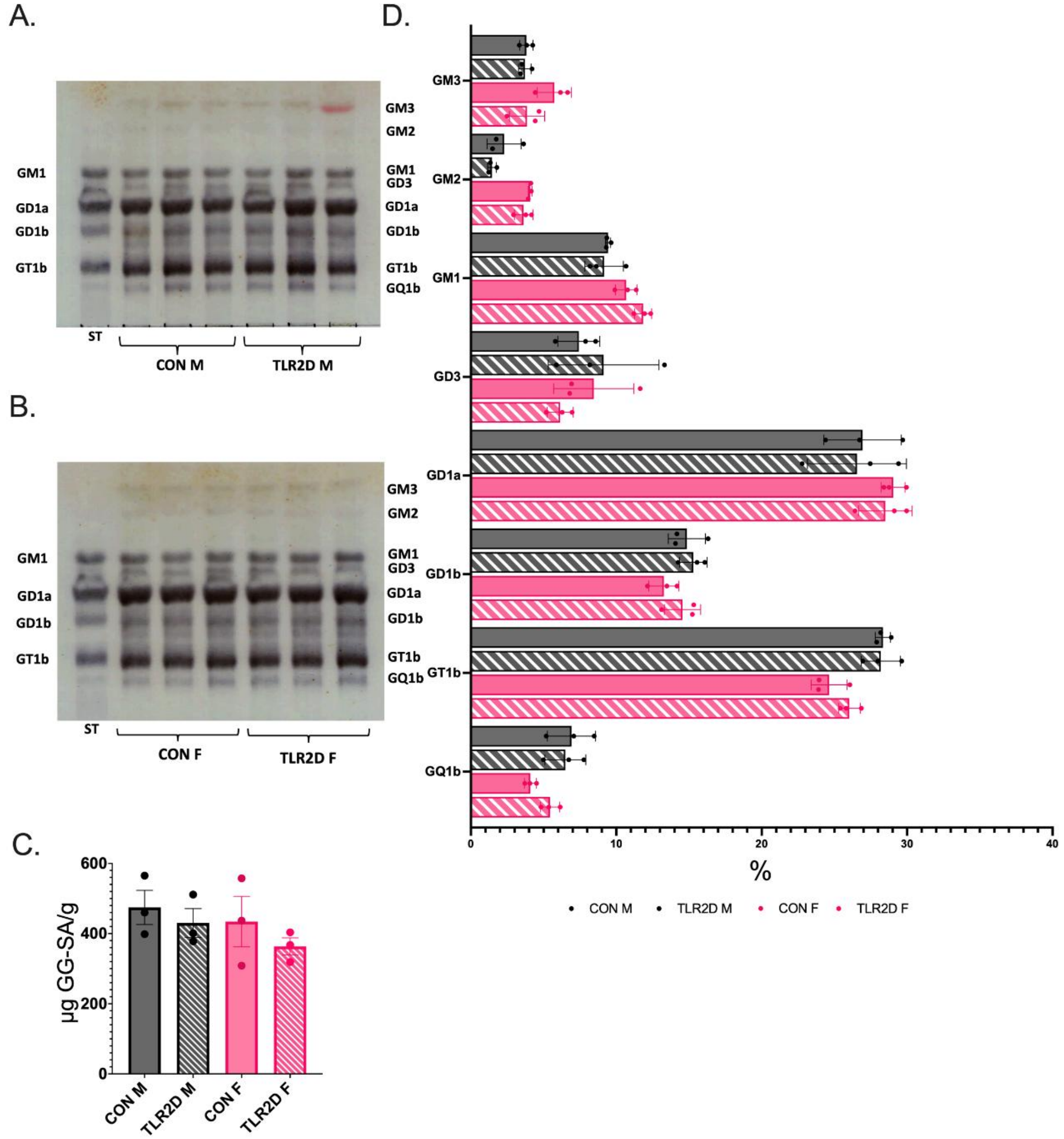

A. High-performance thin layer chromatography (HPTLC) plate showing separation of gangliosides derived from TLR2-deficient male (TLR2D M) and matched control animals (CON M) with identified ganglioside species designated to the right, ST = standard mixture of gangliosides; n = 3 animals/group; B. HPTLC plate showing separation of gangliosides derived from TLR2-deficient female (TLR2D F) and matched control animals (CON F) with identified ganglioside species designated to the right, ST = standard mixture of gangliosides; n = 3 animals/group; C. Proportion of ganglioside species separated by HPTLC and quantified among all groups. Data is shown as mean ± SEM, n = 3 animals/group; D. Quantitative determination of ganglioside-bound sialic acid content among all analyzed groups. Data is shown as mean ± SEM, n = 3 animals/group.

Supplementary Figure 3. (-) NanoESI IMS CID MS/MS of the $[M-2H^+]^{2-}$ at m/z 643.403 corresponding to GM3(d18:1/26:2) species detected in the IMS MS of brain tissue sample derived from control male mice.

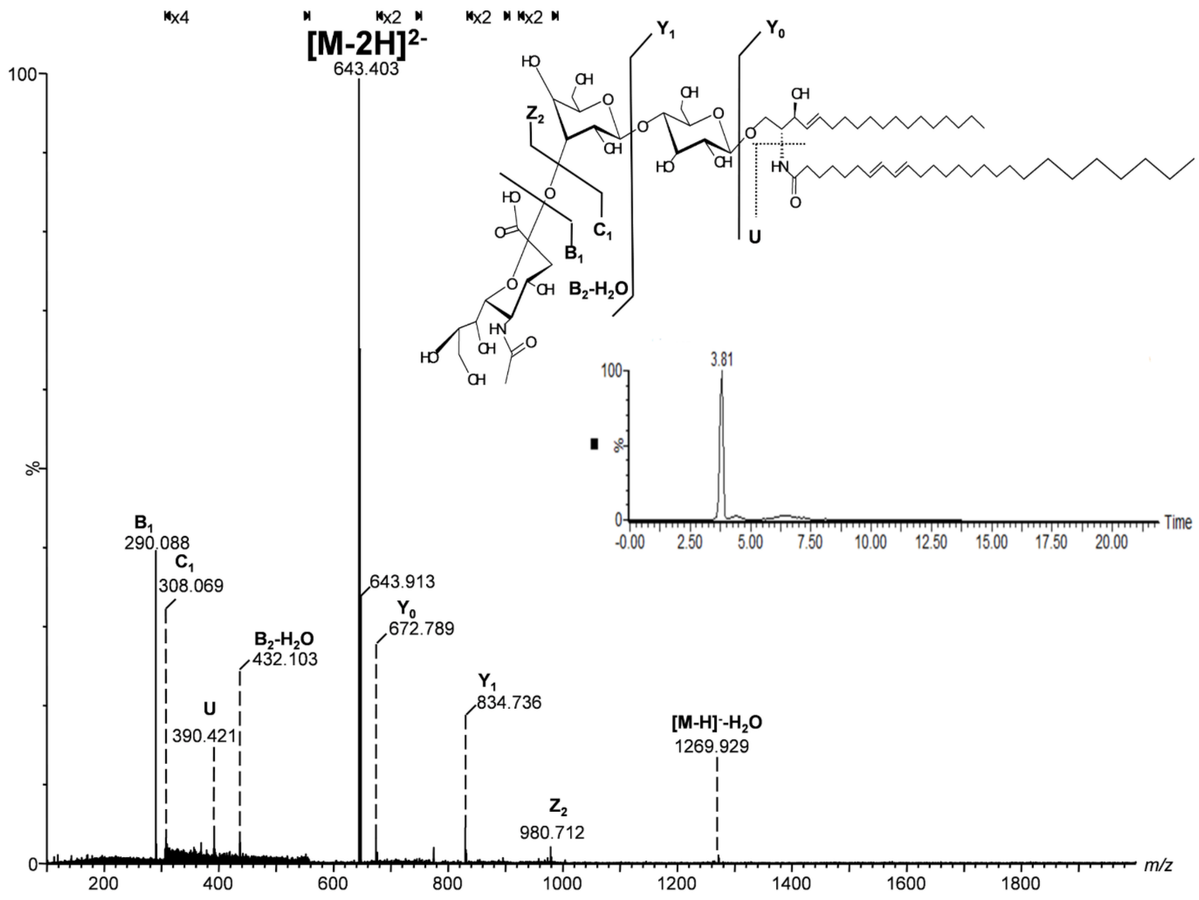

CID at variable collision energy within (30-60) eV; acquisition 150 scans; inserts: the fragmentation scheme and the drift time distribution for the ion at *m/z* 643.403 fragmented by CID.

Supplementary Figure 4. (-) NanoESI IMS CID MS/MS of the $[M-2H^{+}]^{2-}$ at m/z 651.827 corresponding to GM2(d18:1/13:4) species detected in the IMS MS of brain tissue sample derived from control female mice.

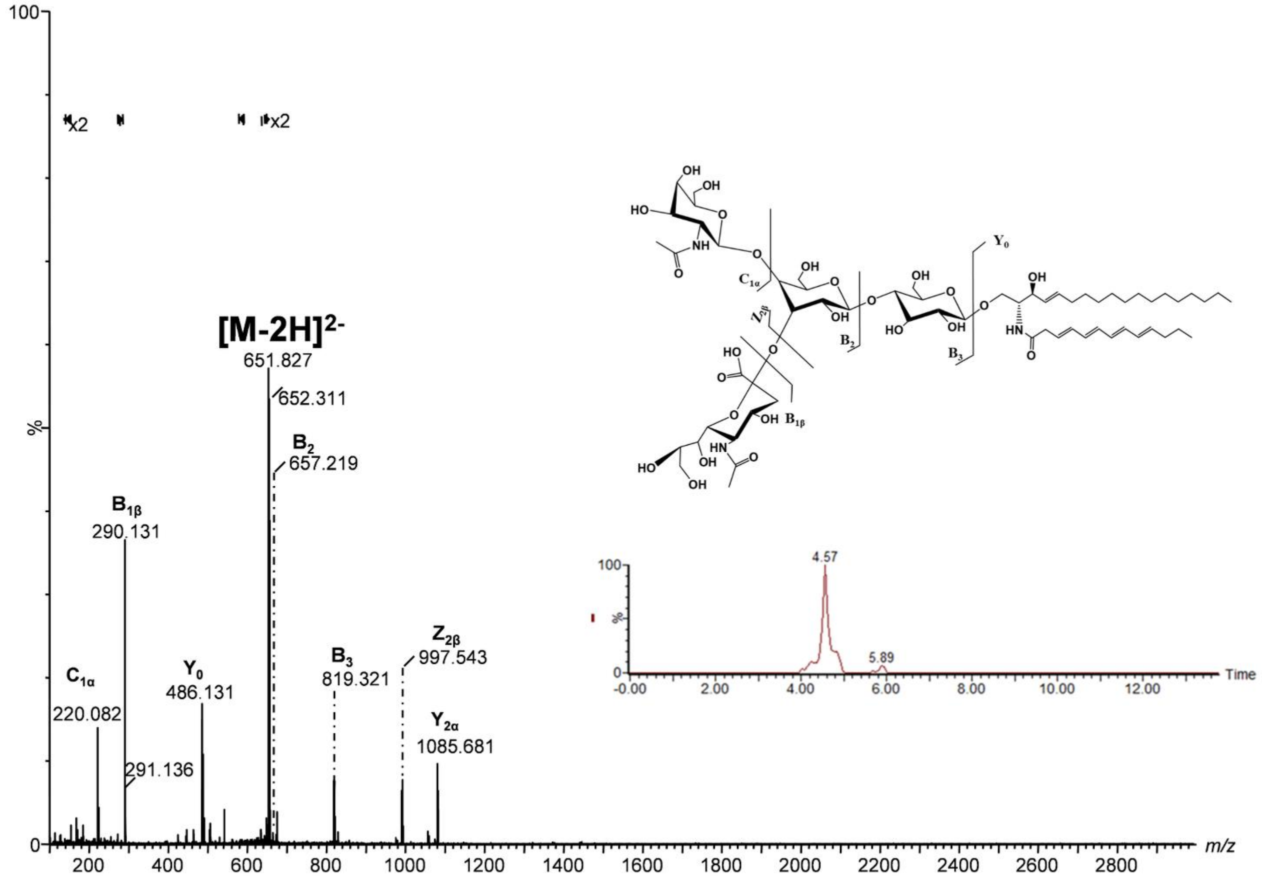

CID at variable collision energy within (30-60) eV; acquisition 150 scans; inserts: the fragmentation scheme and the drift time distribution for the ion at *m/z* 651.827 fragmented by CID.

Supplementary Figure 5. Representative depiction of Western blot confirmation of succesful isolation of lipid rafts, as evidenced by the pertinent localization of lipid raft and non-lipid rafts specific markers.

A.

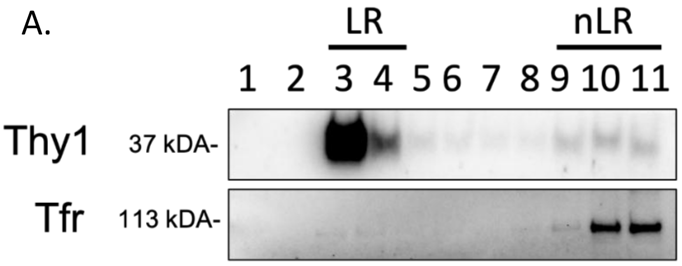

B.

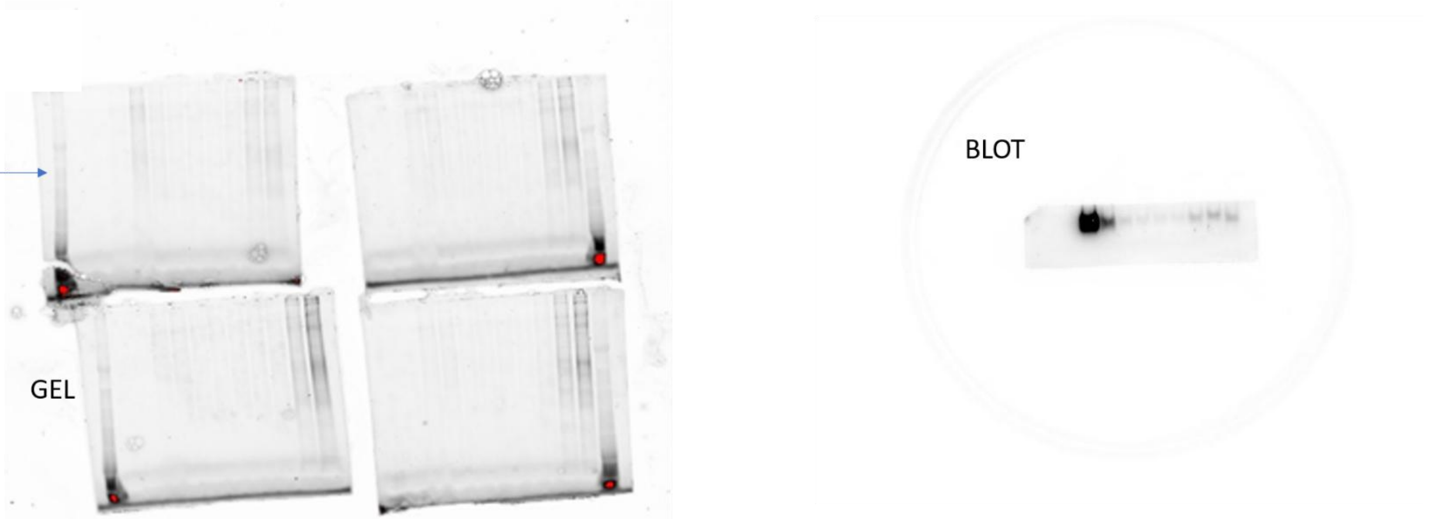

C.

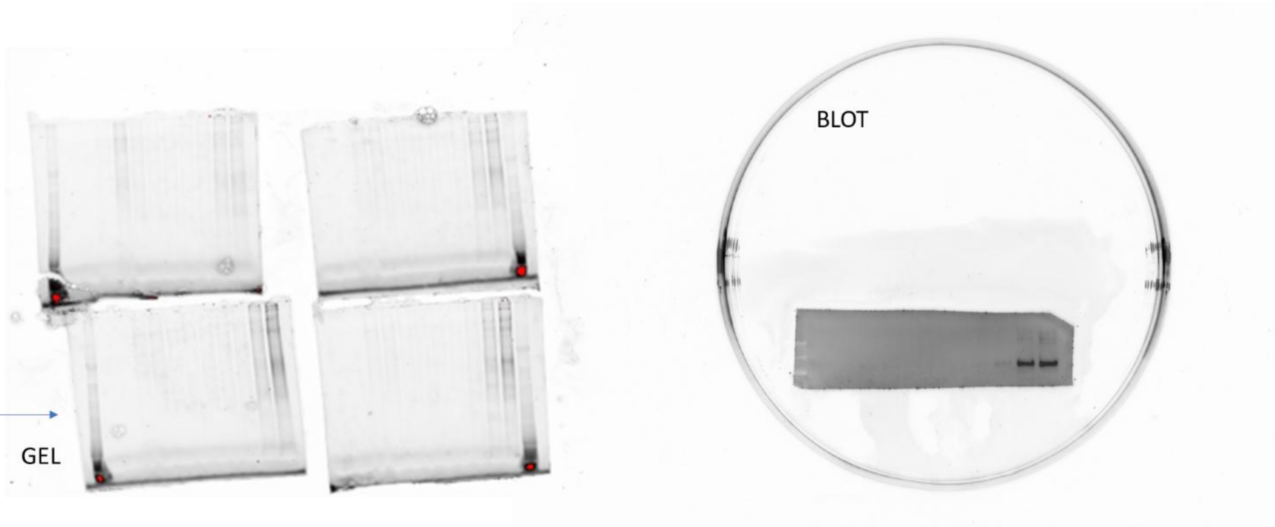

A. Western blot confirmation of Thy1, a lipid raft (LR) marker, within the fractions 2 and 4, and of Tfr, a non-lipid raft (nLR) marker within the fractions 9-11; B. Uncropped gel and blot image of the Thy 1 separation and immunoreactivity detection; C. uncropped gel and blot image of Tfr separation and immunoreactivity detection. Arrows in B. and C. point to the expected size of the protein LR and nLR markers separated by gel-electrophoresis.

Supplementary Figure 6 (Uncropped blot images corresponding to Figure 7.B.). Representative dot-blots of 4 major brain ganglioside species (GM1, GD1a, GD1b, GT1b) in membrane fractions upon membrane sub-fractionation derived from brain tissue of TLR2 deficient male (TLR2D M) and female (TLR2D F) mice, and matched controls (CON M, CON F) -

GM1

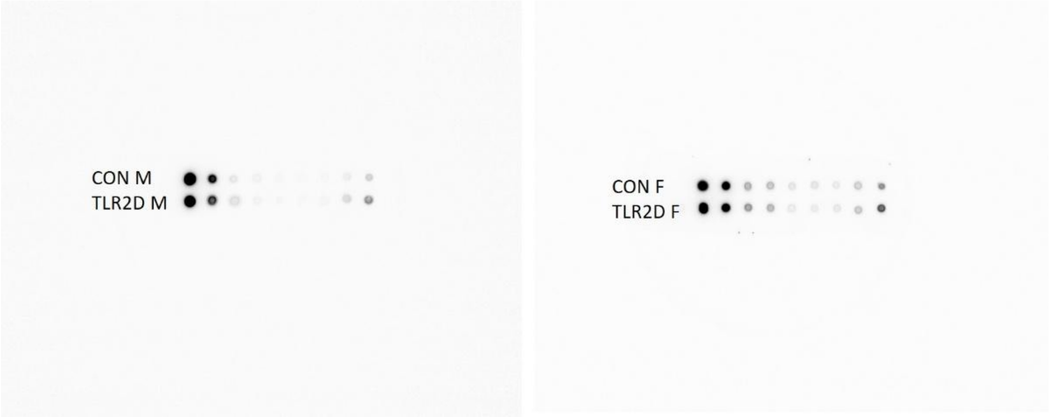

GD1a

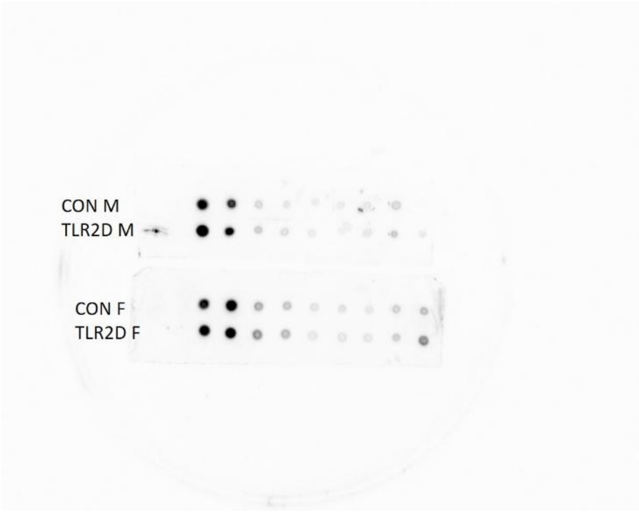

GD1b

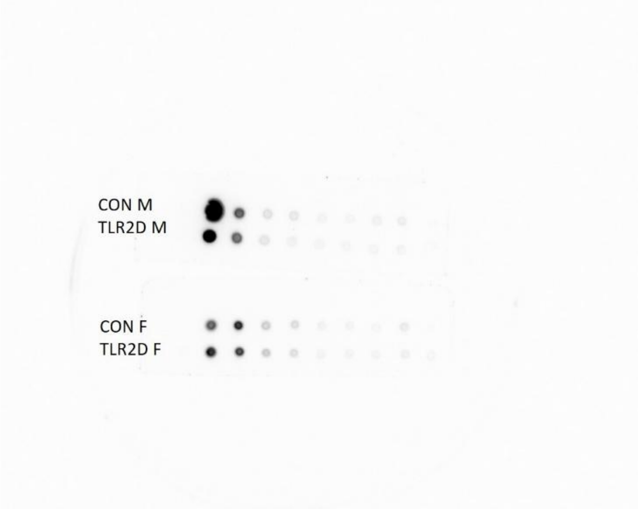

GT1b

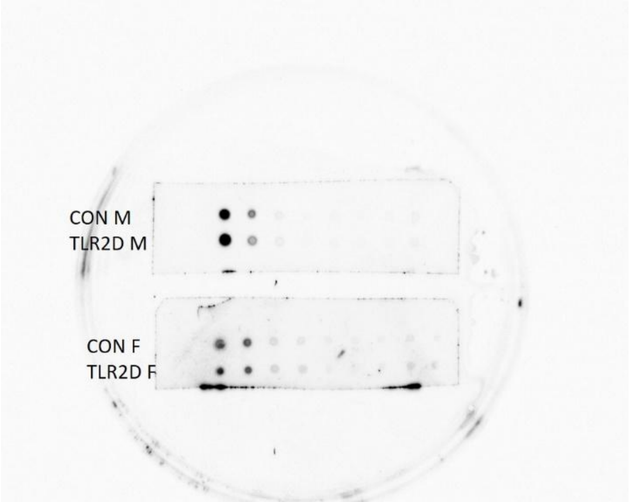

II. SUPPLEMENTARY TABLES

Supplementary Table 1. Assignment of major ionic species detected in CON-F and TLR2D-F by (-) nanoESI IMS MS

m/z theor	*m/z*exp TLR2D-F	ppm TLR2D-F	*m/z*exp CON-F	ppm CON-F	Proposed Structure	Molecular Ion
438,776	438,779	6,85			GA3 (d18:1/16:2)	[M-3H+Na]2-
456,824	456,826	4,39			GA3 (d18:1/20:1)	[M-2H+]2-
525,929			525,934	9,52	GT4 (d18:1/17:3)	$[M-3H^+]^{3-}$
539,628			539,619	16,70	O-Ac-GM1 (d18:0/22:2)	$[M-H_2O-3H^+]3^-$
546,611			546,608	5,49	GD2 (d18:1/16:2)	$[M-3H^+]^{3-}$
556,626			556,619	12,59	GD2(d18:1/18:1)	$[M-3H^+]^{3-}$
562,881			562,871	17,79	GA3 (t18:0/29:0)	$[M-5H^+ +3Na^+]^{2-}$
562,340	562,339	1,78			GM3 (d18:0/14:0)	[M-2H+]2-
569,348	569,344	7,03			GM3 (d18:0/15:0)	[M-2H+]2-
570,982			570,972	17,54	Fuc GM1(d18:1/21:0)	$[M-H_2O-3H^+]3^-$
577,625			577,615	17,33	Fuc GM1(d18:1/20:3)	[M-4H+Na]3-
603,782	603,788	9,95	603,788	9,95	GQ1 (d18:1/18:0)	[M-4H+]4-
610,790	610,795	8,20	610,785	8,20	GQ1 (d18:1/20:0)	[M-4H+]4-
614,285	614,291	9,77	614,292	11,40	GQ1 (t18:1/20:1)	[M-4H+]4-
621,302	621,303	1,61	621,309	11,27	GQ1 (d18:1/23:0)	[M-4H+]4-
624,806	624,809	4,81	624,803	4,81	GQ1 (d18:1/24:0)	[M-4H+]4-
631,814	631,819	7,92	631,812	3,17	GQ1 (d18:0/26:0)	[M-4H+]4-
636,671	636,668	4,72			O-Ac-GT3 (d18:0/24:0)	[M-4H+Na]3-
639,31	639,318	12,52	639,308	3,13	Fuc GT3(t 18:0/16:0)	[M-4H+Na]3-
643,411	643,419	12,44	643,419	12,44	GM3 (d18:1/26:2)	[M-2H+]2-
650,67			650,676	9,23	Fuc GT3(t 18:0/20:0)	[M-3H+]3-
657,862	657,864	3,04			GM2 (t18:0/12:0)	[M-2H+]2-
657,325			657,317	12,18	Fuc GT3(t 18:1/20:0)	[M-4H+Na]3-
666,855	666,847	12,01			GM2 (d18:1/13:0)	[M-3H++Na+]2-
673,864	673,852	17,83	673,858	8,92	GM2 (d18:1/16:3)	$[M-2H^+]^{2-}$
679,863	679,869	8,84	679,857	8,84	GM2 (d18:1/15:1)	$[M-3H^+ +Na^+]^{2-}$
683,656	683,648	11,71			GT1 (d18:1/14:0)	$[M-H_2O-3H^+]^{3-}$
693,672	693,665	10,10	693,682	14,43	GT1 (d18:0/16:0)	$[M-H_2O-3H^+]^{3-}$
699,004	699,015	15,74	699,015	15,74	GT1 (d18:1/16:0)	$[M-3H^+]^{3-}$
699,676			699,681	7,15	GT1 (d18:0/16:0)	$[M-3H^+]^{3-}$
703,004	703,014	14,22			GT1 (d18:1/17:1)	$[M-3H^+]^{3-}$
703,663	703,672	12,80			GT1 (t18:1/16:1)	$[M-3H^+]^{3-}$
707,676	707,669	9,90	707,681	7,07	GT1 (d18:1/18:1)	$[M-3H^+]^{3-}$
708,347	708,345	2,82	708,335	16,95	GT1 (d18:1/18:0)	[M-3H+]3-
713,007	713,004	4,21	713,013	8,42	GT1 (t18:1/18:1)	$[M-3H^+]^{3-}$
713,659			713,668	12,62	GT1 (d18:1/16:0)	$[M-5H^{++2Na}]^{3-}$
717,019	717,011	11,16	717,027	11,16	GT1 (d18:1/20:1)	$[M-3H^+]^{3-}$

717,691	717,671	27,89	717,683	11,16	GT1 (d18:1/20:0)	[M-3H$^+$]$^{3-}$
721,679	721,669	13,87	721,681	2,77	GT1 (t18:1/20:2)	[M-3H$^+$]$^{3-}$
722,351	722,342	12,47	722,362	15,24	GT1 (t18:1/20:1)	[M-3H$^+$]$^{3-}$
726,363	726,372	12,40	726,375	16,53	GT1 (d18:1/22:1)	[M-3H$^+$]$^{3-}$
727,035	727,042	9,63	727,042	9,63	GT1 (d18:1/22:0)	[M-3H$^+$]$^{3-}$
731,023			731,017	8,21	GT1 (t18:1/22:2)	[M-3H$^+$]^{3}
731,695	731,701	8,21	731,689	8,21	GT1 (t18:1/22:1)	[M-3H$^+$]^{3}
734,912	734,922	13,62	734,909	4,09	GD3 (d18:1/18:0)	[M-2H$^+$]$^{2-}$
735,707	735,702	6,80			GT1 (d18:1/24:1)	[M-3H$^+$]$^{3-}$
736,367	736,374	9,51	736,374	9,51	*O*-Ac GT1(d18:1/21:0)	[M-3H$^+$]$^{3-}$
740,893	740,894	1,35			GD3 (t18:1/18:2)	[M-2H$^+$]$^{2-}$
741,044	741,039	6,75	741,051	9,45	GT1 (d18:1/25:0)	[M-3H$^+$]$^{3-}$
741,919	741,914	6,75			GD3 (d18:1/19:0)	[M-2H$^+$]$^{2-}$
743,917	743,926	12,11			GD3 (t18:0/18:0)	[M-H_2O-2H$^+$]$^{2-}$
745,903	745,913	13,42			GD3 (d18:1/18:0)	[M-3H$^+$+Na$^+$]$^{2-}$
745,723	745,734	14,77	745,734	14,77	GT1 (d18:1/26:0)	[M-3H$^+$]$^{3-}$
747,919	747,91	12,05	747,918	1,34	GD3 (d18:1/20:1)	[M-2H$^+$]$^{2-}$
748,927	748,938	14,71			GD3 (d18:1/20:0)	[M-2H$^+$]$^{2-}$
750,394	750,401	9,33			GT1 (d18:1/27:0)	[M-3H$^+$]$^{3-}$
755,69	755,702	15,89			Fuc-GT1 (d18:1/18:2)	[M-3H$^+$]$^{3-}$
757,914	757,916	2,64	757,904	13,21	GM1 (d18:1/16:0)	[M-2H$^+$]$^{2-}$
764,362	764,375	17,02			Fuc-GT1 (d18:1/20:3)	[M-3H$^+$]$^{3-}$
765,93	765,92	13,07			GM1 (d18:0/17:0)	[M-2H$^+$]$^{2-}$
768,362	768,368	7,81	768,373	14,32	Fuc-GT1 (d18:1/21:4)	[M-3H$^+$]$^{3-}$
770,365	770,376	14,29			Fuc-GT1 (t18:1/20:2)	[M-3H$^+$]$^{3-}$
771,93	771,941	14,27	771,928	2,59	GM1 (d18:1/18:0)	[M-2H$^+$]$^{2-}$
775,951	775,955	5,16			GD3 (d18:1/24:1)	[M-2H$^+$]$^{2-}$
776,004	776,01	7,73			GQ1 (d18:1/12:2)	[M-3H$^+$]$^{3-}$
789,365	789,367	2,53	789,357	10,14	GD2(d18:1/12:5)	[M-2H$^+$]$^{2-}$
790,02	790,03	12,66			GQ1 (d18:1/15:2)	[M-3H$^+$]$^{3-}$
793,962	793,949	16,39	793,972	12,61	GM1(d18:0/21:0)	[M-2H$^+$]$^{2-}$
795,364	795,353	13,84			GQ1 (d18:1/16:1)	[M-3H$^+$]$^{3-}$
800,036	800,025	13,75			GQ1 (d18:1/17:1)	[M-3H$^+$]$^{3-}$
801,412	801,417	6,24			GD2 (d18:1/13:0)	[M-2H$^+$]$^{2-}$
801,379	801,387	9,99			GQ1 (d18:0/17:0)	[M-3H$^+$]$^{3-}$
801,965	801,958	8,74			GD3 (d18:1/26:0)	[M-3H$^+$+Na$^+$]$^{2-}$
802,973	802,962	13,72			GD3 (d18:0/26:0)	[M-3H$^+$+Na$^+$]$^{2-}$
804,707	804,712	6,22	804,712	6,22	GQ1 (d18:1/18:1)	[M-3H$^+$]$^{3-}$
805,379	805,368	13,66	805,387	9,94	GQ1 (d18:1/18:0)	[M-3H$^+$]$^{3-}$

806,404	806,409	6,20			GD2 (d18:1/14:3)	$[M-2H^+]^{2-}$
805,379					GQ1 (d18:1/18:0)	$[M-3H^+]^{3-}$
806,951	806,949	2,48			*O*-Ac GM1(d18:1/20:0)	$[M-2H^+]^{2-}$
807,973	807,968	6,20	807,982	11,15	*O*-Ac GM1(d18:0/20:0)	$[M-2H^+]^{2-}$
810,051	810,064	16,05	810,064	16,05	GQ1 (d18:1/19:0)	$[M-3H^+]^{3-}$
811,948	811,938	12,33			GD3 (d18:1/26:1)	$[M-4H^++2Na^+]^{2-}$
812,956	812,948	9,85			GD3 (d18:1/26:0)	$[M-4H^++2Na^+]^{2-}$
813,379	813,368	13,53			GQ1 (d18:1/20:2)	$[M-3H^+]^{3-}$
813,412	813,419	8,61			GD2 (d18:1/15:2)	$[M-2H^+]^{2-}$
814,051	814,062	13,51	814,059	9,83	GQ1 (d18:1/20:1)	$[M-3H^+]^{3-}$
814,42	814,416	4,91			GD2 (d18:0/15:1)	$[M-2H^+]^{2-}$
814,711			814,7150	4,91	*O*-Ac-GQ1 (d18:1/17:0)	$[M-3H^+]^{3-}$
818,723	818,732	11,00	818,7370	17,11	GQ1 (d18:1/21:1)	$[M-3H^+]^{3-}$
819,383	819,392	10,99	819,372	13,43	O-Ac-GQ1 (d18:1/18:0)	$[M-3H^+]^{3-}$
819,383	819,375	9,77			O-Ac-GQ1 (d18:1/18:0)	$[M-3H^+]^{3-}$
820,714	820,705	10,98	820,708	7,32	O-Ac-GQ1 (t18:0/17:0)	$[M-3H^+]^{3-}$
822,723			822,731	9,73	GQ1(d18:1/22:2)	$[M-3H^+]^{3-}$
824,055	824,049	7,28	824,045	12,14	O-Ac-GQ1 (d18:1/19:0)	$[M-3H^+]^{3-}$
822,436	822,444	9,73			GD2 (d18:1/16:0)	$[M-2H^+]^{2-}$
827,395			827,401	7,26	GQ1 (d18:1/23:2)	$[M-3H^+]^{3-}$
828,067			828,077	12,08	GQ1 (d18:1/23:1)	$[M-3H^+]^{3-}$
828,738	828,729	10,87	828,746	9,66	GQ1 (d18:1/23:0)	$[M-3H^+]^{3-}$
832,727	832,737	12,02	832,717	12,02	GQ1 (t18:1/23:2)	$[M-3H^+]^{3-}$
833,428	833,435	8,40	833,435	8,40	GD2 (d18:1/18:3)	$[M-2H^+]^{2-}$
833,411	833,423	14,41			GQ1(t18:0/24:0)	$[M-H_2O-3H^+]3^-$
834,436	834,444	9,59			GD2 (d18:1/18:2)	$[M-2H^+]^{2-}$
835,394	835,386	9,58			GQ1(d18:1/23:1)	$[M-4H^++Na^+]3^-$
835,443			835,453	11,98	GD2 (d18:1/18:1)	$[M-2H^+]^{2-}$
836,066			836,075	10,77	GQ1(d18:1/23:0)	$[M-4H^++Na^+]3^-$
836,451	836,465	16,75			GD2 (d18:1/18:0)	$[M-2H^+]^{2-}$
838,082	838,097	17,90	838,092	11,93	GQ1 (d18:1/25:0)	$[M-3H^+]^{3-}$
841,443	841,453	11,89	841,452	10,70	GD2 (d18:1/19:2)	$[M-2H^+]^{2-}$
842,451	842,436	17,81	842,462	13,06	GD2 (d18:1/19:1)	$[M-2H^+]^{2-}$
842,082	842,091	10,69			GQ1 (d18:1/26:1)	$[M-3H^+]^{3-}$
843,722	843,73	9,49	843,728	7,12	GP2 (d18:1/17:0)	$[M-3H^+]^{3-}$
843,41	843,419	10,68			GD2 (d18:1/16:1)	$[M-4H^++2Na^+]^{2-}$
844,449	844,437	14,22			GD2 (t18:1/18:0)	$[M-2H^+]^{2-}$
845,456	845,444	14,20			GD2 (t18:0/18:0)	$[M-2H^+]^{2-}$
846,741	846,751	11,82	846,731	11,82	GQ1 (t18:0/24:0)	$[M-4H^++Na^+]3^-$

846,434	846,448	16,55			GD2 (d18:1/18:1)	$[M-3H^+ +Na^+]^{2-}$
847,442	847,452	11,81			GD2 (d18:1/18:0)	$[M-3H^+ +Na^+]^{2-}$
848,451	848,45	1,18	848,463	14,15	GD2 (d18:1/20:2)	$[M-2H^+]^{2-}$
847,414			847,427	15,35	*O*-Ac GQ1(t18:0/24:0)	$[M-H_2O-3H^+]3^-$
848,451	848,457	7,08			GD2 (d18:1/20:2)	$[M-2H^+]^{2-}$
850,467	850,456	12,94			GD2 (d18:1/20:0)	$[M-2H^+]^{2-}$
851,475	851,475	0,00			GD2 (d18:0/20:0)	$[M-2H^+]^{2-}$
852,433	852,439	7,04	852,44	8,22	GD2 (d18:0/17:0)	$[M-4H^+ +2Na^+]^{2-}$
854,451	854,447	4,68			GD2 (d18:1/21:3)	$[M-2H^+]^{2-}$
855,459	855,469	11,70			GD2 (d18:1/21:2)	$[M-2H^+]^{2-}$
856,467	856,477	11,68	856,475	9,35	GD2 (d18:1/21:1)	$[M-2H^+]^{2-}$
857,425	857,437	14,00			GD2 (d18:1/18:1)	$[M-4H^+ +2Na^+]^{2-}$
858,483	858,478	5,83			GD2 (d18:0/21:0)	$[M-2H^+]^{2-}$
859,442	859,449	8,15			GD2 (d18:1/20:2)	$[M-3H^+ +Na^+]^{2-}$
860,45	860,446	4,65	860,44	11,63	GD2 (d18:1/20:1)	$[M-3H^+ +Na^+]^{2-}$
862,467	862,456	12,76			GD2 (d18:1/22:2)	$[M-2H^+]^{2-}$
870,483			870,485	2,30	GD2 (d18:1/23:1)	$[M-2H^+]^{2-}$
876,425	876,432	7,99	876,432	7,99	GP2 (d18:1/24:0)	$[M-3H^+]^{3-}$
878,442	878,456	15,95			GT3 (d18:0/16:0)	$[M-3H^+ +Na^+]^{2-}$
878,461	878,469	9,11			(CH_3COO^-)-GD2 (d18:0/18:0)	$[M-3H^+ +Na^+]^{2-}$
879,452	879,447	5,69			GT3 (d18:1/18:1)	$[M-2H^+]^{2-}$
895,483	895,473	11,17	895,498	16,76	GT3 (d18:0/20:0)	$[M-2H^+]^{2-}$
897,444	897,439	5,57			GD1 (t18:1/14:0)	$[M-2H^+]^{2-}$
903,462	903,478	17,72	903,478	17,72	GD1 (d18:1/16:0)	$[M-2H^+]^{2-}$
907,468	907,469	1,10			GD1 (d18:1/18:1)	$[M-H_2O-2H^+]^{2-}$
908,472	908,482	11,01			GD1 (d18:1/18:0)	$[M-H_2O-2H^+]^{2-}$
909,48	909,485	5,50			*O*-Ac-GT3 (d18:0/19:0)	$[M-2H^+]^{2-}$
910,452	910,456	4,40	910,469	18,68	GD1 (t18:1/16:1)	$[M-2H^+]^{2-}$
910,452	910,449	3,30			GD1 (t18:1/16:1)	$[M-2H^+]^{2-}$
911,43	911,421	9,88	911,429	1,10	GD1 (d18:1/16:3)	$[M-3H^+ +Na^+]^{2-}$
912,437	912,447	10,96			GD1 (d18:1/16:2)	$[M-3H^+ +Na^+]^{2-}$
913,445	913,436	9,86			GD1 (d18:1/16:1)	$[M-3H^+ +Na^+]^{2-}$
916,47	916,482	13,10	916,481	12,01	GD1 (d18:1/18:1)	$[M-2H^+]^{2-}$
916,47	916,486	17,47			GD1 (d18:1/18:1)	$[M-2H^+]^{2-}$
917,478	917,481	3,27	917,461	18,54	GD1 (d18:1/18:0)	$[M-2H^+]^{2-}$
920,491	920,487	4,35			GT3(d18:1/24:2)	$[M-2H^+]^{2-}$
924,467	924,477	10,82	924,457	10,82	GD1 (t18:1/18:1)	$[M-2H^+]^{2-}$
925,475	925,466	9,73	925,477	2,16	GD1 (t18:1/18:0)	$[M-2H^+]^{2-}$
930,485	930,492	7,53	930,492	7,53	GD1 (d18:1/20:1)	$[M-2H^+]^{2-}$

931,493	931,502	9,67	931,502	9,67	GD1 (d18:1/20:0)	$[M-2H^+]^{2-}$
931,493	931,502	9,67			GD1 (d18:1/20:0)	$[M-2H^+]^{2-}$
935,476			935,461	16,04	GD1 (d18:1/19:0)	$[M-3H^++Na^+]^{2-}$
938,471	938,477	6,40	938,483	12,79	O-*Ac*-GT3 (d18:1/22:2)	$[M-3H^++Na^+]^{2-}$
938,501	938,504	3,20			GD1 (d18:1/21:0)	$[M-2H^+]^{2-}$
940,467			940,4750	8,51	GD1 (d18:0/18:0)	$[M-4H^++2Na^+]^{2-}$
942,486			942,498	12,74	GD1 (d18:1/22:3)	$[M-2H^+]^{2-}$
944,501	944,505	4,24			GD1 (d18:1/22:1)	$[M-2H^+]^{2-}$
945,509	945,515	6,35	945,512	3,17	GD1 (d18:1/22:0)	$[M-2H^+]^{2-}$
946,517	946,526	9,51			GD1 (d18:0/22:0)	$[M-2H^+]^{2-}$
947,463	947,471	8,45	947,455	8,45	GD1 (t18:1/18:1)	$[M-4H^++2Na^+]^{2-}$
948,463	948,472	9,49			GD1 (t18:1/18:0)	$[M-4H^++2Na^+]^{2-}$
949,474	949,47	4,21	949,482	8,43	GD1 (t18:1/20:1)	$[M-3H^++Na^+]^{2-}$
952,499	952,509	10,50	952,509	10,50	O-Ac-GD1 (d18:1/20:0)	$[M-2H^+]^{2-}$
952,499	952,509	10,50			GD1 (t18:1/22:1)	$[M-2H^+]^{2-}$
953,506	953,507	1,05			GD1 (t18:1/22:0)	$[M-2H^+]^{2-}$
955,51	955,503	7,33			O-Ac-GT3 (d18:0/24:0)	$[M-3H^++Na^+]^{2-}$
956,501	956,516	15,69	956,496	5,23	GD1 (d18:1/24:3)	$[M-2H^+]^{2-}$
957,491	957,506	15,67			GD1 (t18:1/23:3)	$[M-2H^+]^{2-}$
958,479			958,481	2,09	(CH_3COO^-)-GD1 (d18:1/18:0)	$[M-3H^++Na^+]^{2-}$
959,525	959,531	6,26	959,512	13,56	GD1 (d18:1/24:0)	$[M-2H^+]^{2-}$
959,506	959,492	14,60			GD1 (t18:1/23:1)	$[M-2H^+]^{2-}$
961,532	961,524	8,32			GD1 (t18:0/23:0)	$[M-2H^+]^{2-}$
964,468	964,476	8,30			GD1 (d18:1/22:3)	$[M-4H^++2Na^+]^{2-}$
965,487	965,485	2,07			(CH_3COO^-)-GD1 (d18:1/19:0)	$[M-3H^++Na^+]^{2-}$
966,507			966,511	3,11	*O*-Ac GD1(d18:1/22:0)	$[M-2H^+]^{2-}$
967,504	967,507	3,10			Fuc-GT3 (d18:1/20:0)	$[M-2H^+]^{2-}$
971,469	971,481	12,36	971,472	3,09	Fuc-GT3 (t18:1/18:1)	$[M-3H^++Na^+]^{2-}$
972,495	972,503	8,23	972,482	13,37	(CH3COO-)-GD1 (d18:1/20:0)	$[M-3H^++Na^+]^{2-}$
973,484	973,473	11,31	973,498	14,39	Fuc-GT3 (t18:0/18:0)	$[M-3H^++Na^+]^{2-}$
974,512	974,524	12,32			Fuc-GT3 (d18:1/21:0)	$[M-2H^+]^{2-}$
975,501			975,492	9,23	Fuc-GT3 (t18:1/20:0)	$[M-2H^+]^{2-}$
976,516	976,513	3,07	976,5230	7,17	GD1(d18:1/25:1)	$[M-3H^++Na^+]^{2-}$
981,52	981,519	1,02	981,511	9,17	Fuc-GT3 (d18:1/22:0)	$[M-2H^+]^{2-}$
982,527			982,536	9,16	Fuc-GT3 (d18:0/22:0)	$[M-2H^+]^{2-}$
987,5	987,495	5,07	987,509	9,12	Fuc-GT3 (t18:0/20:0)	$[M-3H^++Na^+]^{2-}$
988,491					Fuc-GD1 (d18:1/18:2)	$[M-2H^+]^{2-}$
1008,505	1008,512	6,94	1008,495	9,92	Fuc-GD1(d18:1/19:0)	$[M-3H^++Na^+]^{2-}$

1014,506			1014,505	0,99	Fuc-GD1(d18:1/20:1)	$[M-3H^{+}+Na^{+}]^{2-}$
1017,955	1017,949	5,90			GT1 (d18:1/12:3)	$[M-2H^{+}]^{2-}$
1017,955	1017,945	9,83	1017,941	13,77	GT1 (d18:1/12:3)	$[M-2H^{+}]^{2-}$
1018,963	1018,979	15,72	1018,955	7,86	GT1 (d18:1/12:2)	$[M-2H^{+}]^{2-}$
1031,971	1031,973	1,94			GT1 (d18:1/14:3)	$[M-2H^{+}]^{2-}$
1032,979	1032,984	4,84	1032,965	13,57	GT1 (d18:1/14:2)	$[M-2H^{+}]^{2-}$
1039,544	1039,548	3,85			Fuc-GD1 (t18:1/24:1)	$[M-2H^{+}]^{2-}$
1039,967			1039,953	12,42	GT1 (t18:1/12:0)	$[M-3H^{+}+Na^{+}]^{2-}$
1041,559	1041,567	7,68	1041,547	11,53	Fuc-GD1 (t18:0/24:0)	$[M-2H^{+}]^{2-}$
1047,982	1047,968	13,37	1047,969	12,42	GT1 (t18:0/13:0)	$[M-3H^{+}+Na^{+}]^{2-}$
1051,542	1051,539	2,85	1051,559	16,18	Fuc-GD1 (t18:1/24:0)	$[M-3H^{+}+Na^{+}]^{2-}$
1054,003			1054,011	7,59	GT1 (d18:1/17:2)	$[M-2H^{+}]^{2-}$
1062,018	1062,028	9,42	1062,01	7,53	GT1 (d18:1/18:1)	$[M-2H^{+}]^{2-}$
1063,025	1063,039	13,17	1063,039	13,17	GT1 (d18:1/18:0)	GT1 (d18:1/18:0)
1070,034			1070,028	5,61	GT1 (d18:1/19:0)	$[M-2H^{+}]^{2-}$
1071,042	1071,039	2,80			GT1 (d18:0/19:0)	$[M-2H^{+}]^{2-}$
1073,009	1073,015	5,59			GT1 (d18:1/18:1)	$[M-3H^{+}+Na^{+}]^{2-}$
1074,007			1074,018	10,24	GT1 (d18:1/18:0)	$[M-3H^{+}+Na^{+}]^{2-}$
1077,041	1077,035	5,57	1077,052	10,21	GT1 (d18:1/20:0)	$[M-2H^{+}]^{2-}$
1082,014	1082,026	11,09	1082,006	7,39	GT1 (t18:1/18:0)	$[M-3H^{+}+Na^{+}]^{2-}$
1084,049	1084,058	8,30	1084,033	14,76	GT1 (d18:1/21:0)	$[M-2H^{+}]^{2-}$
1091,057	1091,042	13,75	1091,048	8,25	GT1 (d18:1/22:0)	$[M-2H^{+}]^{2-}$
1095,04	1095,059	17,35	1095,059	17,35	GT1 (d18:1/21:0)	$[M-3H^{+}+Na^{+}]^{2-}$
1096,031			1096,051	18,25	GT1 (t18:1/22:3)	$[M-2H^{+}]^{2-}$
1097,007	1097,017	9,12	1097,017	9,12	GT1 (d18:1/20:2)	$[M-4H^{+}+2Na^{+}]^{2-}$
1104,065	1104,072	6,34			GT1 (d18:1/24:1)	$[M-2H^{+}]^{2-}$
1105,072	1105,064	7,24	1105,084	10,86	GT1 (d18:1/24:0)	$[M-2H^{+}]^{2-}$
1112,08	1112,068	10,79	1112,078	1,80	GT1 (d18:1/25:0)	$[M-2H^{+}]^{2-}$
1117,072	1117,091	17,01			GT1 (d18:1/26:2)	$[M-2H^{+}]^{2-}$
1118,08	1118,069	9,84			GT1 (d18:1/26:1)	$[M-2H^{+}]^{2-}$
1124,08	1124,062	16,01	1124,073	6,23	(CH_3COO^{-})-GT1 (d18:1/20:3)	$[M-4H^{+}+2Na^{+}]^{2-}$
1131,044	1131,039	4,42			Fuc-GT1 (t18:0/16:0)	$[M-2H^{+}]^{2-}$
1140,039			1140,026	11,40	Fuc-GT1 (d18:1/19:3)	$[M-2H^{+}]^{2-}$
1141,047	1141,038	7,89			Fuc-GT1 (d18:1/19:2)	$[M-2H^{+}]^{2-}$
1150,568	1150,583	13,04	1150,548	17,39	GQ2(t18:0/20:0)	$[M-2H^{+}]^{2-}$
1162,07	1162,063	6,02			Fuc-GT1 (d18:1/22:2)	$[M-2H^{+}]^{2-}$
1164,086			1164,067	16,32	Fuc-GT1 (d18:1/22:0)	$[M-2H^{+}]^{2-}$
1207,565	1207,552	10,77			GQ1(d18:1/18:1)	$[M-2H^{+}]^{2-}$
1208,573	1208,564	7,45	1208,554	15,73	GQ1(d18:1/18:0)	$[M-2H^{+}]^{2-}$

1222,589	1222,594	4,09			GQ1(d18:1/20:0)	$[M-2H^{+}]^{2-}$
1230,555	1230,544	8,94	1230,549	4,88	GQ1 (d18:1/18:0)	$[M-4H^{+}+2Na^{+}]^{2-}$
1248,603	1248,617	11,22	1248,617	11,22	GQ1(d18:0/22:0)	$[M-3H^{+}+Na^{+}]^{2-}$
1250,62			1250,639	15,20	GQ1(d18:1/24:0)	$[M-2H^{+}]^{2-}$
1262,621	1262,639	14,26	1262,639	14,26	GQ1(d18:1/26:2)	$[M-2H^{+}]^{2-}$
1264,636	1264,643	5,54			GQ1(d18:1/26:0)	$[M-2H^{+}]^{2-}$
1269,628	1269,633	3,94	1269,617	8,67	GQ1(d18:1/27:2)	$[M-2H^{+}]^{2-}$
1283,644	1283,647	2,34	1283,625	14,81	GQ1(d18:1/29:2)	$[M-2H^{+}]^{2-}$

Supplementary Table 2. Assignment of major ionic species detected in CON-M and TLR2D-M by (-) nanoESI IMS MS

m/z theor	*m/z* exp TLR2D-M	ppm TLR2D-M	*m/z* exp CON-M	ppm CON-M	Proposed structure	Molecular ion
439,784	439,789	11,39	439,792	18,22	GA3 (d18:1/16:1)	$[M-3H+Na]^{2-}$
447,781	447,783	4,47	447,786	11,19	GA3 (t18:1/16:1)	$[M-3H+Na]^{2-}$
454,789			454,786	6,61	GA3 (t18:1/17:1)	$[M-3H+Na]^{2-}$
456,824			456,819	10,96	GA3 (d18:1/20:1)	$[M-2H+]^{2-}$
525,932	525,924	15,24			GT4 (d18:1/17:3)	$[M-3H]^{3-}$
530,284	530,281	5,66			O-Ac-GM1 (d18:0/20:2)	$[M-H_2O-3H^+]^{3-}$
539,628	539,619	16,70			O-Ac-GM1 (d18:0/22:2)	$[M-H_2O-3H^+]^{3-}$
551,025			551,019	10,89	GT1(d18:1/24:2)	$[M-4H^+]^{4-}$
562,881	562,876	8,90	562,879	3,56	GA3 (t18:0/29:0)	$[M-5H^++3Na^+]^{2-}$
584,313			584,303	17,12	Fuc GM1 (d18:0/21:0)	$[M-4H+Na]^{3-}$
603,782	603,79	13,27			GQ1 (d18:1/18:0)	$[M-4H^+]^{4-}$
610,790	610,8	16,39			GQ1 (d18:1/20:0)	$[M-4H^+]^{4-}$
614,285	614,296	17,92			GQ1 (t18:1/20:1)	$[M-4H^+]^{4-}$
621,302	621,309	11,27			GQ1 (d18:1/23:0)	$[M-4H^+]^{4-}$
624,806	624,819	20,83			GQ1 (d18:1/24:0)	$[M-4H^+]^{4-}$
631,814	631,826	18,78			GQ1 (d18:0/26:0)	$[M-4H^+]^{4-}$
636	636,277	7,86			GT2(d18:1/13:3)	$[M-4H^+ +Na^+]^{3-}$
643,411	643,418	10,89	643,403	12,44	GM3 (d18:1/26:2)	$[M-2H^+]^{2-}$
650,969	650,966	4,62			GT2 (d18:0/14:0)	$[M-5H^++2Na^+]^{3-}$
651,833			651,827	9,22	GM2 (d18:1/13:4)	$[M-2H^+]^{2-}$
657,862	657,852	15,22	657,858	6,09	GM2 (t18:0/12:0)	$[M-2H^+]^{2-}$
663,880	663,890	15,08	663,887	10,56	GM2 (d18:0/14:0)	$M-2H^+]^{2-}$
671,878	671,876	2,98	671,868	14,90	GM2 (t18:0/14:0)	$[M-2H^+]^{2-}$
673,864			673,859	7,43	GM2 (d18:1/16:3)	$[M-2H^+]^{2-}$
679,458	679,446	17,67	679,452	8,84	GM3 (d18:1/31:0)	$[M-2H^+]^{2-}$
690,849			690,843	8,70	GD3 (d18:1/12:2)	$[M-2H^+]^{2-}$
704,335			704,334	1,42	GT1(t18:1/16:0)	$[M-3H^+]^{3-}$
704,865	704,863	2,84			GD3 (d18:1/14:2)	$[M-2H^+]^{2-}$
706,880			706,876	5,67	GD3 (d18:1/14:0)	$[M-2H^+]^{2-}$
707,676	707,671	7,07			GT1 (d18:1/18:1)	$[M-3H^+]^{3-}$
708,347	708,351	5,65	708,349	2,82	GT1 (d18:1/18:0)	$[M-3H+]^{3-}$
713,019	713,013	8,42	713,011	11,22	GT1 (d18:1/19:0)	$[M-3H^+]$3-
713,658	713,649	12,62			GT1 (d18:1/16:0)	$[M-5H^++2Na^+]^{3-}$
713,676	713,671	7,01			GT1 (t18:1/18:0)	$[M-3H^+]^{3-}$
714,351	714,355	5,60			GT1 (t18:0/18:0)	$[M-3H^+]^{3-}$
717,691	717,697	8,37	717,699	11,16	GT1 (d18:1/20:0)	$[M-3H^+]^{3-}$
720,896			720,886	13,89	GD3 (d18:1/16:0)	$[M-2H^+]^{2-}$
722,351	722,349	2,77	722,342	12,47	GT1 (t18:1/20:1)	$[M-3H^+]^{3-}$
727,035	727,042	9,63			GT1 (d18:1/22:0)	$[M-3H^+]^{3-}$
731,695	731,701	8,21	731,688	9,58	GT1 (t18:1/22:1)	$[M-3H^+]^{3-}$
734,912	734,919	9,54	734,915	4,09	GD3 (d18:1/18:0)	$[M-2H^+]^{2-}$
735,707	735,703	5,44			O-Ac-GT1 (d18:0/22:0)	$[M-H2O-3H^+]^{3-}$

736,379	736,374	6,79	736,371	10,87	GT1 (d18:1/24:0)	$[M-3H^+]^{3-}$
737,051			737,044	9,50	GT1 (d18:0/24:0)	$[M-3H^+]^{3-}$
741,710	741,718	10,80			GT1 (t18:1/24:0)	$[M-3H^+]^{3-}$
745,334	745,328	8,05			GQ2 (t18:1/16:3)	$[M-3H^+]^{3-}$
749,038	749,035	4,01			GT1 (t18:1/24:0)	$[M-4H^++Na]^{3-}$
757,914	757,904	13,21	757,926	15,85	GM1 (d18:1/16:0)	$[M-2H^+]^{2-}$
768,362	768,371	11,72			Fuc-GT1 (d18:1/21:4)	$[M-3H^+]^{3-}$
770,377			770,381	5,19	Fuc-GT1 (d18:1/21:1)	$[M-3H^+]^{3-}$
771,948	771,956	10,38			GD3 (t18:0/22:0)	$[M-2H^+]^{2-}$
776,381	776,376	6,44			Fuc-GT1 (t18:1/21:0)	$[M-3H^+]^{3-}$
789,93	789,927	3,80	789,923	8,87	GM1 (d18:1/21:3)	$[M-2H^+]^{2-}$
793,397	793,387	12,61	793,406	11,35	GD2(d18:1/12:1)	$[M-2H^+]^{2-}$
799,352	799,346	7,51			GQ1 (t18:1/16:3)	$[M-3H^+]^{3-}$
800,404	800,401	3,75			GD2(d18:1/13:1)	$[M-2H^+]^{2-}$
800,707	800,717	12,50			GQ1 (d18:0/17:1)	$[M-3H^+]^{3-}$
801,379	801,383	4,99	801,384	6,24	GQ1 (d18:0/17:0)	$[M-3H^+]^{3-}$
804,707	804,711	4,98			GQ1 (d18:1/18:1)	$[M-3H^+]^{3-}$
805,397	805,407	12,42	805,392	6,21	GQ1 (d18:1/18:0)	$[M-3H^+]^{3-}$
811,948	811,941	8,63	811,94	9,86	GD3 (d18:1/26:1)	$[M-4H^++Na^+]^{2-}$
812,707	812,697				GQ1 (d18:1/18:0)	$[M-4H^++Na^+]^{3-}$
814,723	814,733	12,29	814,719	4,91	GQ1 (d18:1/20:0)	$[M-3H^+]^{3-}$
814,420	814,431	13,51			GD2 (d18:0/15:1)	$[M-2H^+]^{2-}$
819,395	819,402	8,55	819,387	9,77	GQ1 (d18:1/21:0)	$[M-3H^+]^{3-}$
819,412	819,422	12,21			GD2 (d18:1/16:1)	$[M-2H^+]^{2-}$
820,419	820,408	13,41	820,413	7,32	GD2 (d18:0/14:0)	$[M-3H^++Na^+]^{2-}$
820,714	820,721	8,54			O-Ac-GQ1 (t18:0/17:0)	$[M-3H^+]^{3-}$
824,055	824,043	14,56			O-Ac-GQ1 (d18:1/19:0)	$[M-3H^+]^{3-}$
826,710	826,706	4,84			O-Ac-GQ1 (d18:1/18:0)	$[M-4H^++Na^+]^{3-}$
827,446			827,441	6,05	GD2 (d18:1/18:0)	$[M-H2O-2H^+]^{2-}$
828,739	828,744	6,04	828,731	9,66	GQ1 (d18:1/23:0)	$[M-3H^+]^{3-}$
829,398			829,385	15,68	GQ1 (d18:1/22:0)	$[M-3H^+]^{3-}$
833,411	833,413	2,40	833,407	4,80	GQ1 (d18:1/24:0)	$[M-3H^+]^{3-}$
834,082			834,068	16,79	GQ1 (d18:0/24:0)	$[M-3H^+]^{3-}$
835,443	835,438	5,99			GD2 (d18:1/18:1)	$[M-2H^+]^{2-}$
837,411			837,414	3,58	GQ1 (d18:1/25:1)	$[M-3H^+]^{3-}$
838,754			838,758	4,77	GQ1 (d18:0/25:0)	$[M-3H^+]^{3-}$
840,454			840,461	8,33	GD2 (d18:1/20:1)	$[M-H2O-2H^+]^{2-}$
842,082	842,072	11,88	842,086	4,75	GQ1 (d18:1/26:1)	$[M-3H^+]^{3-}$
846,741	846,751	11,82			GQ1 (t18:0/24:0)	$[M-4H^++Na^+]^{3-}$
852,433	852,444	12,91			GD2 (d18:0/17:0)	$[M-4H^++2Na^+]^{2-}$
860,45	860,439	12,79			GD2 (d18:1/20:1)	$[M-3H^++Na^+]^{2-}$
895,483	895,493	11,17	895,489	6,70	GT3 (d18:0/20:0)	$[M-2H^+]^{2-}$
900,456	900,468	13,33	900,448	8,89	GT3 (t18:0/18:0)	$[M-3H^++Na^+]^{2-}$
903,480	903,491	12,18	903,490	11,07	GT3 (t18:0/20:0)	$[M-2H^+]^{2-}$
907,468	907,457	12,13	907,471	3,31	GD1 (d18:1/18:1)	$[M-H_2O-2H^+]^{2-}$

910,470	910,476	6,59	910,478	8,79	GD1 (d18:1/17:0)	$[M-2H^{+}]^{2-}$
911,459	911,462	3,29	911,449	10,98	GD1 (t18:1/16:0)	$[M-2H^{+}]^{2-}$
912,437	912,444	7,68			GD1 (d18:1/16:2)	$[M-3H^{+}+Na^{+}]^{2-}$
914,471	914,467	4,38			GT3 (t18:0/20:0)	$[M-3H^{+}+Na^{+}]^{2-}$
916,470	916,482	13,10	916,473	3,28	GD1 (d18:1/18:1)	$[M-2H^{+}]^{2-}$
917,478	917,488	10,91	917,471	7,63	GD1 (d18:1/18:0)	$[M-2H^{+}]^{2-}$
924,467	924,457	10,82	924,463	4,33	GD1 (t18:1/18:1)	$[M-2H^{+}]^{2-}$
925,475	925,473	2,16	925,468	7,57	GD1 (t18:1/18:0)	$[M-2H^{+}]^{2-}$
926,483	926,496	14,04			GD1 (t18:0/18:0)	$[M-2H^{+}]^{2-}$
928,469	928,475	6,47			GD1 (d18:1/18:0)	$[M-3H^{+}+Na^{+}]^{2-}$
930,504	930,509	5,38			O-Ac-GT3 (d18:0/22:0)	$[M-2H^{+}]^{2-}$
931,493	931,492	1,07	931,489	4,30	GD1 (d18:1/20:0)	$[M-2H^{+}]^{2-}$
935,458	935,461	3,21	935,445	13,90	GD1 (t18:1/18:1)	$[M-3H^{+}+Na^{+}]^{2-}$
938,501	938,506	5,33			GD1 (d18:1/21:0)	$[M-2H^{+}]^{2-}$
937,474			937,468	6,40	GD1 (t18:0/18:0)	$[M-3H^{+}+Na^{+}]^{2-}$
940,467			940,455	12,77	GD1 (d18:0/18:0)	$[M-4H^{+}+2Na^{+}]^{2-}$
941,476			941,473	3,19	GD1 (d18:1/20:1)	$[M-3H^{+}+Na^{+}]^{2-}$
942,484			942,475	9,55	GD1 (d18:1/20:0)	$[M-3H^{+}+Na^{+}]^{2-}$
944,501	944,505	4,24			GD1 (d18:1/22:1)	$[M-2H^{+}]^{2-}$
945,509	945,502	7,41	945,499	10,58	GD1 (d18:1/22:0)	$[M-2H^{+}]^{2-}$
946,517	946,506	11,63	946,502	15,86	GD1 (d18:0/22:0)	$[M-2H^{+}]^{2-}$
947,458			947,453	5,28	GD1 (t18:1/20:3)	$[M-3H+Na]^{2-}$
948,463			948,462	1,05	GD1 (t18:1/18:0)	$[M-4H^{+}+2Na^{+}]^{2-}$
949,492	949,502	10,54	949,487	5,27	GD1 (d18:1/21:0)	$[M-3H^{+}+Na^{+}]^{2-}$
952,517	952,525	8,40	952,518	1,05	GD1 (d18:1/23:0)	$[M-2H^{+}]^{2-}$
955,492			955,486	6,28	GD1 (d18:1/22:1)	$[M-3H^{+}+Na^{+}]^{2-}$
956,500	956,496	4,18	956,492	8,37	GD1 (d18:1/22:0)	$[M-3H^{+}+Na^{+}]^{2-}$
957,478			957,465	13,58	O-Ac-GT3 (d18:1/23:1)	$[M-4H^{+}+2Na^{+}]^{2-}$
958,517	958,507	10,44	958,526	9,39	GD1 (d18:1/24:1)	$[M-2H^{+}]^{2-}$
960,483			960,478	5,21	O-Ac-GT3 (t18:0/22:0)	$[M-4H^{+}+2Na^{+}]^{2-}$
964,468			964,459	9,34	GD1 (d18:1/22:3)	$[M-4H^{+}+2Na^{+}]^{2-}$
965,475	965,48	5,18	965,473	2,07	GD1 (d18:0/20:0)	$[M-5H^{+}+3Na^{+}]^{2-}$
966,483	966,477	6,21	966,473	10,35	GD1 (d18:1/22:1)	$[M-4H^{+}+2Na^{+}]^{2-}$
967,504	967,503	1,03			Fuc-GT3 (d18:1/20:0)	$[M-2H^{+}]^{2-}$
970,516	970,521	5,15			GD1 (d18:0/24:1)	$[M-3H^{+}+Na^{+}]^{2-}$
971,523	971,512	11,33			GD1 (d18:0/24:0)	$[M-3H^{+}+Na^{+}]^{2-}$
972,495			972,487	8,23	(CH3COO-) GD1 (d18:1/20:0)	$[M--3H^{+}+Na^{+}]^{2-}$
972,974			972,972	2,06	GT2 (d18:0/15:0)	[M-3H++Na+]2-
973,484	973,494	10,28	973,478	6,17	Fuc-GT3 (t18:0/18:0)	$[M-3H^{+}+Na^{+}]^{2-}$
976,516			976,519	3,07	GD1(d18:1/25:1)	$[M-3H^{+}+Na^{+}]^{2-}$
979,491			979,484	7,15	GD1 (d18:1/24:2)	$[M-4H^{+}+2Na^{+}]^{2-}$
980,494			980,493	1,02	GD1 (d18:1/24:1)	$[M-4H^{+}+2Na^{+}]^{2-}$
981,52	981,511	9,17			Fuc-GT3 (d18:1/22:0)	$[M-2H^{+}]^{2-}$
982,509			982,51	1,02	Fuc-GT3 (t18:1/21:0)	[M-2H+]2-
987,483	987,477	6,08	987,474	9,12	Fuc-GD1 (d18:1/18:3)	[M-2H+]2-
988,491	988,483	8,10	988,482	9,11	Fuc-GD1 (d18:1/18:2)	[M-2H+]2-

989,499			989,487	12,13	Fuc-GD1 (d18:1/18:1)	[M-2H+]2-
990,496			990,488	8,08	Fuc-GT3 (d18:1/24:5)	[M-2H+]2-
991,982	991,978	4,04	991,972	10,09	GT2 (d18:1/18:1)	$[M-3H^{+}+Na^{+}]^{2-}$
992,504			992,506	2,02	Fuc-GD1 (t18:0/17:0)	$[M-2H^{+}]^{2-}$
992,990			992,995	5,04	GT2 (d18:1/18:0)	$[M-3H^{+}+Na^{+}]^{2-}$
993,520	993,527	7,05			Fuc-GT3 (d18:1/24:2)	$[M-2H^{+}]^{2-}$
998,990			998,985	5,01	GT2 (d18:1/19:1)	$[M-3H^{+}+Na^{+}]^{2-}$
999,481	999,474	7,01			$(CH_3COO\text{-})_2$ GD1 (d18:1/18:0)	$[M-4H^{+}+2Na^{+}]^{2-}$
1001,535	1001,521	13,99			Fuc-GT3(d18:1/25:1)	$[M-2H^{+}]^{2-}$
1002,543			1002,538	4,99	Fuc-GT3(d18:1/25:0)	$[M-2H^{+}]^{2-}$
1004,523	1004,533	9,96	1004,536	12,95	Fuc-GD1(d18:1/20:0)	$[M-2H^{+}]^{2-}$
1006,520	1006,535	14,91			Fuc-GD1(t18:0/19:0)	$[M-2H^{+}]^{2-}$
1007,498	1007,505	6,95			Fuc-GD1(d18:1/19:1)	$[M--3H^{+}+Na^{+}]^{2-}$
1008,51	1008,565	59,52			Fuc-GD1(d18:1/19:0)	$[M--3H^{+}+Na^{+}]^{2-}$
1013,528	1013,526	1,97	1013,522	5,92	Fuc-GD1(t18:0/20:0)	$[M-2H^{+}]^{2-}$
1014,51	1014,515	8,88	1014,518	11,83	Fuc-GD1(d18:1/20:1)	$[M-3H^{+}+Na^{+}]^{2-}$
1015,514			1015,523	8,87	Fuc-GD1(d18:1/20:0)	$[M-3H^{+}+Na^{+}]^{2-}$
1016,522	1016,517	4,92	1016,513	8,86	Fuc-GD1(d18:0/20:0)	$[M--3H^{+}+Na^{+}]^{2-}$
1017,955	1017,958	2,95	1017,964	8,85	GT1 (d18:1/12:3)	$[M-2H^{+}]^{2-}$
1018,963	1018,961	1,96	1018,967	3,93	GT1 (d18:1/12:2)	$[M-2H^{+}]^{2-}$
1018,981	1018,985	3,93			Fuc-GT2 (d18:1/13:1)	$[M-2H^{+}]^{2-}$
1021,514			1021,517	2,94	Fuc GD1 d18:1/21:1)	$[M-3H^{+}+Na^{+}]^{2-}$
1024,520	1024,516	3,91			Fuc GD1 (d18:1/22:3)	$[M-2H^{+}]^{2-}$
1026,554	1026,552	1,95			Fuc GD1 (d18:0/23:0)	$[M-2H^{+}]^{2-}$
1027,514	1027,516	1,95			Fuc GD1 (d18:1/22:2)	$[M-3H^{+}+Na^{+}]^{2-}$
1028,522	1028,528	5,84			Fuc GD1(d18:1/22:1)	$[M-3H^{+}+Na^{+}]^{2-}$
1031,971	1031,966	4,85	1031,979	7,76	GT1 (d18:1/14:3)	$[M-2H^{+}]^{2-}$
1033,562	1033,557	4,84			Fuc- GD1 (d18:0/24:0)	$[M-2H^{+}]^{2-}$
1034,994			1034,996	1,93	GT1 (d18:1/14:0)	$[M-2H^{+}]^{2-}$
1040,551	1040,549	1,92	1040,567	15,38	Fuc- GD1 (t18:1/24:0)	$[M-2H^{+}]^{2-}$
1040,994	1040,986	7,69			GT1 (d18:1/15:1)	$[M-2H^{+}]^{2-}$
1041,113	1041,073	38,42			GS2(d18:1/18:2)	$[M-3H^{+}]^{3-}$
1044,554			1044,558	3,83	Fuc- GD1 (d18:1/26:2)	$[M-2H^{+}]^{2-}$
1047,982	1047,973	8,60	1047,965	16,24	GT1 (t18:0/13:0)	$[M-3H^{+}+Na^{+}]^{2-}$
1049,010	1049,021	10,49			GT1 (d18:1/16:0)	$[M-2H^{+}]^{2-}$
1050,554			1050,562	7,62	Fuc GD1 (d18:1/27:3)	$[M-2H^{+}]^{2-}$
1051,562	1051,571	8,56			Fuc GD1 (d18:1/27:2)	$[M-2H^{+}]^{2-}$
1053,577			1053,562	14,25	Fuc GD1(d18:1/27:0)	$[M-2H^{+}]^{2-}$
1055,010	1055,025	14,22			GT1 (d18:1/17:1)	$[M-2H^{+}]^{2-}$
1056,553	1056,543	9,47			Fuc GD1 (d18:1/26:1)	$[M-3H^{+}+Na^{+}]^{2-}$
1062,018	1062,024	5,65	1062,028	9,42	GT1 (d18:1/18:1)	$[M-2H^{+}]^{2-}$
1063,025	1063,035	9,41	1063,031	5,64	GT1 (d18:1/18:0)	$[M-2H^{+}]^{2-}$
1069,561			1069,578	15,90	Fuc GD1 (t18:0/28:0)	$[M-2H^{+}]^{2-}$
1074,018	1074,028	9,31	1074,029	9,32	GT1 (d18:1/20:3)	$[M-2H^{+}]^{2-}$
1077,041	1077,051	9,29	1077,035	5,57	GT1 (d18:1/20:0)	$[M-2H^{+}]^{2-}$

1084,049	1084,053	3,69	1084,032	15,68	GT1 (d18:1/21:0)	$[M-2H^+]^{2-}$
1088,032	1088,027	4,60			GT1 (d18:1/20:0)	$[M-3H^+ +Na^+]^{2-}$
1091,057	1091,048	8,25	1091,042	13,75	GT1 (d18:1/22:0)	$[M-2H^+]^{2-}$
1092,064			1092,074	9,16	GT1 (d18:0/22:0)	$[M-2H^+]^{2-}$
1095,040	1095,069	26,48			GT1 (d18:1/21:0)	$[M-3H^+ +Na^+]^{2-}$
1097,039	1097,042	2,73	1097,025	12,76	GT1 (t18:1/22:2)	$[M-2H^+]^{2-}$
1104,065			1104,048	15,40	GT1 (d18:1/24:1)	$[M-2H^+]^{2-}$
1105,07	1105,084	10,86			GT1 (d18:1/24:0)	$[M-2H^+]^{2-}$
1106,081			1106,074	6,33	GT1 (d18:0/24:0)	$[M-2H^+]^{2-}$
1108,028			1108,015	11,73	GT1 (t18:0/20:0)	$[M-4H^+ +2Na^+]^{2-}$
1109,500	1109,491	8,12			GQ2(d18:1/16:4)	$[M-2H^+]^{2-}$
1111,515	1111,518	2,70	1111,528	11,70	GQ2(d18:1/16:2)	$[M-2H^+]^{2-}$
1118,523	1118,514	8,05			GQ2(d18:1/17:2)	$[M-2H^+]^{2-}$
1119,531			1119,536	4,47	GQ2(d18:1/17:1)	$[M-2H^+]^{2-}$
1126,096			1126,081	13,32	CH_3COO^- GT1(d18:1/20:3)	$[M-3H^+ +2Na^+]^{2-}$
1132,539	1132,533	5,30			GQ2(d18:1/19:2)	$[M-2H^+]^{2-}$
1141,047	1141,038	7,89			Fuc-GT1 (d18:1/19:2)	$[M-2H^+]^{2-}$
1142,570			1142,561	7,88	GQ2(d18:0/20:0)	$[M-2H^+]^{2-}$
1150,57	1150,581	11,30			GQ2(t18:0/20:0)	$[M-2H^+]^{2-}$
1155,062			1155,054	6,93	Fuc GT1 (d18:1/21:2)	$[M-2H^+]^{2-}$
1181,550	1181,547	2,54			GQ1(d18:0/14:0)	$[M-2H^+]^{2-}$
1191,534	1191,527	5,88			GQ1(d18:1/16:3)	$[M-2H^+]^{2-}$
1208,574	1208,589	12,42	1208,563	9,11	GQ1(d18:1/18:0)	$[M-2H^+]^{2-}$
1215,563	1215,557	4,94			GQ1(t18:1/18:1)	$[M-2H^+]^{2-}$
1219,564	1219,567	2,46			GQ1(d18:1/18:0)	$[M-3H^+ +Na^+]^{2-}$
1222,589	1222,573	13,09	1222,582	5,73	GQ1(d18:1/20:0)	$[M-2H^+]^{2-}$
1227,562	1227,574	9,78			GQ1 (t18:1/18:0)	$[M-3H^+ +Na^+]^{2-}$
1229,561	1229,549	9,76	1229,551	8,14	*O*-Ac-GQ1 (d18:1/18:0)	$[M-2H^+]^{2-}$
1235,597	1235,586	8,91			GQ1(d18:0/22:1)	$[M-2H^+]^{2-}$
1236,604			1236,616	9,71	GQ1(d18:1/22:0)	$[M-2H^+]^{2-}$
1243,563	1243,573	8,05	1243,554	7,24	GQ1(d18:1/20:1)	$[M-4H^+ +2Na^+]^{2-}$
1248,603	1248,617	11,22			GQ1(d18:0/22:0)	$[M-3H^+ +Na^+]^{2-}$
1249,612	1249,629	13,61	1249,621	7,21	GQ1 (t18:1/24:0)	$[M-H2O-2H^+]^{2-}$
1264,64	1264,652	12,66	1264,653	13,45	GQ1(d18:1/26:0)	$[M-2H^+]^{2-}$
1269,628			1269,611	13,40	GQ1(d18:1/27:2)	$[M-2H^+]^{2-}$

Supplementary Table 3. Assignment of the fragment ions originating from the $[M-2H^+]^{2-}$ at m/z 917.488 corresponding to the GD1(d18:1/18:0), ie GD1(d18:1/18:0) *m/z* 917.488 $[M-2H^+]^{2-}$ species isolated from KM sample and fragmented by (-) nanoESI IMS CID MS/MS

m/z	Number of negative charges	Fragment Ion
282.287	1	U
290.104	1	$B_{1\alpha}$
308.310	1	$C_{1\alpha}$
364.140	1	$B_{2\beta}$ or $C_3/C_{2\alpha}/B_{1\beta}$ or $C_3/B_{2\alpha}/C_{1\beta}$
470.171	1	$C_3/B_{2\beta}/B_{1\alpha}$
493.185	1	$^{2,4}A_4/B_{1\alpha}/C_{2\beta}$
546.547	1	Z_0
564.562	1	Y_0
581.093	1	$B_{2\alpha}$
655.2485	1	$C_3/C_{1\alpha}/B_{1\beta}$ or $C_3/B_{1\alpha}/C_{1\beta}$
708.618	1	Z_1
726.6248	1	Y_1 or $B_3/C_{2\beta}$
735.422	2	$Y_{2\beta}$
827.732	2	$Z_{3\beta}$
836.829	2	$Y_{3\beta}$
888.532	1	$Y_{2\beta}/B_{2\alpha}$or $B_4/C_{2\beta}$
963.391	1	$C_3/B_{1\beta}$
1073.444	1	$Y_{3\beta}/C_{2\alpha}$ or $Z_{3\beta}/B_{2\alpha}$
1091.776	1	$B_4/C_{1\beta}$ or $Y_{3\beta}/B_{2\alpha}$
1179.770	1	$Y_{2\beta}/B_{1\alpha}$
1253.839	1	$Y_{2\alpha}$
1364.841	1	$Y_{3\beta}/C_{1\alpha}$or $Z_{3\beta}/B_{1\alpha}$
1382.701	1	$Y_{3\beta}/B_{1\alpha}$
1526.922	1	$Z_{3\alpha}$
1544.891	1	$Y_{3\alpha}$

Supplementary Table 4. Summary of differentially expressed genes identified in pairwise comparisons between TLR2-deficient and control mice of both sexes

Group comparison	Total DEGs	Upregulated genes	Downregulated genes
CON F vs. CON M	574	170	404
TLR2D F vs. TLR2D M	314	133	181
CON F vs. TLR2D F	356	103	253
CON M vs. TLR2D M	565	275	290

Data established using DESeq2 and edgeR, applying significance criteria of adjusted p-value < 0.05 and fold-change (FC) ≥ 1.5. DEGs, differentially expressed genes; TLR2D, TLR2-deficient; CON, control; F, female; M, male.

Supplementary Table 5. Gene annotations for genes implicated in ganglioside and cholesterol metabolism shown in Figure 8.A-C, respectively.

Gene symbol	Ensembl ID	Official gene name	Encoded protein	Cellular localization	Biological function
St3gal5	ENSMUSG000 00019863	ST3 beta-galactoside alpha-2,3-sialyltransferase 5	GM3 synthase	Golgi apparatus	Catalyzes synthesis of GM3 from lactosylceramide
B4galt5	ENSMUSG000 00017929	UDP-Gal:betaGlcNAc beta 1,4-galactosyltransferase, polypeptide 5	B-1,4-Galactosyltransferase 5	Golgi apparatus	Glycosphingolipid biosynthesis
B4galt6	ENSMUSG000 00056124	UDP-Gal:betaGlcNAc beta 1,4-galactosyltransferase, polypeptide 6	B-1,4-Galactosyltransferase 6	Golgi apparatus	Involved in lactosylceramide biosynthesis
B4galnt1	ENSMUSG000 00006731	Beta-1,4-N-acetyl-galactosaminyl transferase 1	GM2/GD2 synthase	Golgi apparatus	Catalyzes synthesis of GM2/GD2 gangliosides
St8sia1	ENSMUSG000 00030283	ST8 alpha-N-acetyl-neuraminide alpha-2,8-sialyltransferase 1	GD3 synthase	Golgi apparatus	Catalyzes synthesis of gangliosides GD3 and GT3
St8sia3	ENSMUSG000 00056812	ST8 alpha-N-acetyl-neuraminide alpha-2,8-sialyltransferase 3	Sialyltransferase 8C	Golgi apparatus	Sialylation of gangliosides (e.g. GD1c, GT1a)
St8sia5	ENSMUSG000 00056812	ST8 alpha-N-acetyl-neuraminide alpha-2,8-sialyltransferase 3	Sialyltransferase 8E	Golgi apparatus	Involved in polysialylation of gangliosides
B3galt4	ENSMUSG000 00067370	UDP-Gal:betaGlcNAc beta 1,3-galactosyltransferase, polypeptide 4	B-1,3-Galactosyltransferase 4	Golgi apparatus	Biosynthesis of gangliosides GM1, GD1b, GT1b
Neu2	ENSMUSG000 00079434	Neuraminidase 2	Sialidase-2	Cytoplasm	Hydrolyzes sialic acids from glycolipids and glycoproteins
Neu3	ENSMUSG000 00035239	Neuraminidase 3	Sialidase-3	Plasma membrane	Hydrolyzes sialic acids from gangliosides
Neu4	ENSMUSG000 00034000	Neuraminidase 4	Sialidase-4	Lysosome, Cytosol	Removal of sialic acids from glycoproteins, glycolipids
Gm2a	ENSG0000019 6743	GM2 ganglioside activator	GM2 activator protein	Lysosome	Activates degradation of GM2 ganglioside by β-hexosaminidase A
Psap	ENSMUSG000 00004207	Prosaposin	Prosaposin (precursor of saposins A-D)	Lysosome, Secreted	Glycolipid degradation, neurotrophic functions
Hexa	ENSMUSG000 00025232	Hexosaminidase subunit alpha	Hexosaminidase A subunit	Lysosome	Catalyzes removal of terminal N-acetyl hexosamines in ganglioside GM2 degradation
Glb1	Ensmusg0000 0015129	Galactosidase beta 1	B-galactosidase	Lysosome	Degradation of gangliosides and glycoproteins
Asah1	ENSMUSG000 00031591	N-acylsphingosine amidohydrolase 1	Acid ceramidase	Lysosome	Hydrolyzes ceramide into sphingosine and free fatty acid; key in sphingolipid catabolism

Insig1	ENSMUSG00000045294	Insulin induced gene 1	INSIG1	Endoplasmic reticulum	Retains SCAP/SREBP in ER to inhibit cholesterol biosynthesis
Insig2	ENSMUSG00000003721	Insulin induced gene 2	INSIG2	Endoplasmic reticulum	Negative regulator of cholesterol biosynthesis (SREBP signaling)
Hmgcr	ENSMUSG00000021670	3-hydroxy-3-methylglutaryl-CoA reductase	HMG-CoA reductase	Endoplasmic reticulum	Rate-limiting enzyme in cholesterol biosynthesis
Mvk	ENSMUSG00000041939	Mevalonate kinase	Mevalonate kinase	Cytoplasm	Catalyzes step in mevalonate pathway of cholesterol biosynthesis
Mvd	ENSMUSG00000006517	Mevalonate diphosphate decarboxylase	Diphosphomevalonate decarboxylase (MDDase)	Cytoplasm	Catalyzes step in cholesterol and isoprenoid biosynthesis
Sqle	ENSMUSG00000022351	Squalene monooxygenase	Squalene epoxidase	Endoplasmic reticulum	Converts squalene to 2,3-oxidosqualene in sterol biosynthesis
Hsd17b7	ENSMUSG00000026675	3-keto-steroid reductase/17-beta-hydroxysteroid dehydrogenase 7	17-beta-hydroxysteroid dehydrogenase 7	Endoplasmic reticulum, cytosol	Catalyzes step in cholesterol biosynthesis and steroid metabolism
Dhcr7	ENSMUSG00000058454	7-dehydrocholesterol reductase	7-dehydrocholesterol reductase	Endoplasmic reticulum	Converts 7-dehydrocholesterol to cholesterol
Dhcr24	ENSMUSG00000034926	3-beta-hydroxysterol delta-24 reductase	24-dehydrocholesterol reductase	Endoplasmic reticulum	Reduces desmosterol to cholesterol
Npc1	ENSMUSG00000024413	NPC intracellular cholesterol transporter 1	Niemann-Pick C1 protein	Late endosome, lysosome	Cholesterol trafficking from lysosomes

The table lists gene symbols, Ensembl gene IDs, official gene names, encoded protein products, cellular localization, and a summary of functional roles. Information was compiled from publicly available databases (UniProt, MGI).
